# Expanded use of benzamidines in the Groebke–Blackburn–Bienaymé synthesis of imidazoles and their biological evaluation

**DOI:** 10.3762/bjoc.22.108

**Published:** 2026-09-30

**Authors:** Kamryn Walker, Sydney Rivas, Erik Tillman-Smith, Kayla McLeod, Jeanee Bullock, Jacqueline Smith

**Affiliations:** 1 Howard University, Department of Chemistry, 525 College Street, Washington DC 10059, USA; 2 Bowie State University, 14000 Jericho Park Dr, Bowie MD 20715, USA

**Keywords:** acyclic amidine, cancer therapeutics, cytotoxicity, Groebke–Blackburn–Bienaymé reaction, imidazoles, valosin-containing protein

## Abstract

Heterocyclic compounds are often considered privileged scaffolds because many bioactive molecules contain these moieties. Several inhibitors of the valosin-containing protein (VCP, also known as p97), a target for cancer therapeutics, contain 5 or 6-membered heterocyclic cores. With the rise of resistance among these VCP inhibitors, we propose a new class of heterocycles as potential cancer therapeutics. The Groebke–Blackburn–Bienaymé (GBB) reaction is a classic method to produce imidazole compounds. In our earlier study, we showed that acyclic amidines can effectively produce trisubstituted imidazoles known as benzyl imine tricyclic imidazoles (BITIs) via this multicomponent reaction. Herein, we report the first systematic evaluation of *para*-substituted benzamidines produced via the microwave-assisted GBB reaction, which enables the tuning of trisubstituted imidazole compounds and their application as VCP inhibitors. Several of the newly developed compounds inhibit VCP activity, with one amidinopyridine derivative showing low-micromolar, cancer-selective cytotoxicity. This establishes a new scaffold for VCP inhibitor development.

## Introduction

Valosin-containing protein (VCP), also known as p97, is a key ATPase in the ubiquitin proteasome system (UPS) and has emerged as a therapeutic target for various cancers [1–5]. Like other targets in the UPS, the inhibition of VCP is expected to cause lethality by causing an accumulation of misfolded proteins in the cell [6–8]. Several heterocyclic compounds have been identified as VCP inhibitors [9–12]. Small molecules that inhibit the ATPase function of VCP prevent the mechanical action of various VCP-containing complexes and therefore inhibit the UPS, activate the unfolded protein response (UPR), and induce apoptosis. However, ATPase inhibitors often lack selectivity or lose effectiveness due to mutations [13–15].

**Figure 1:**
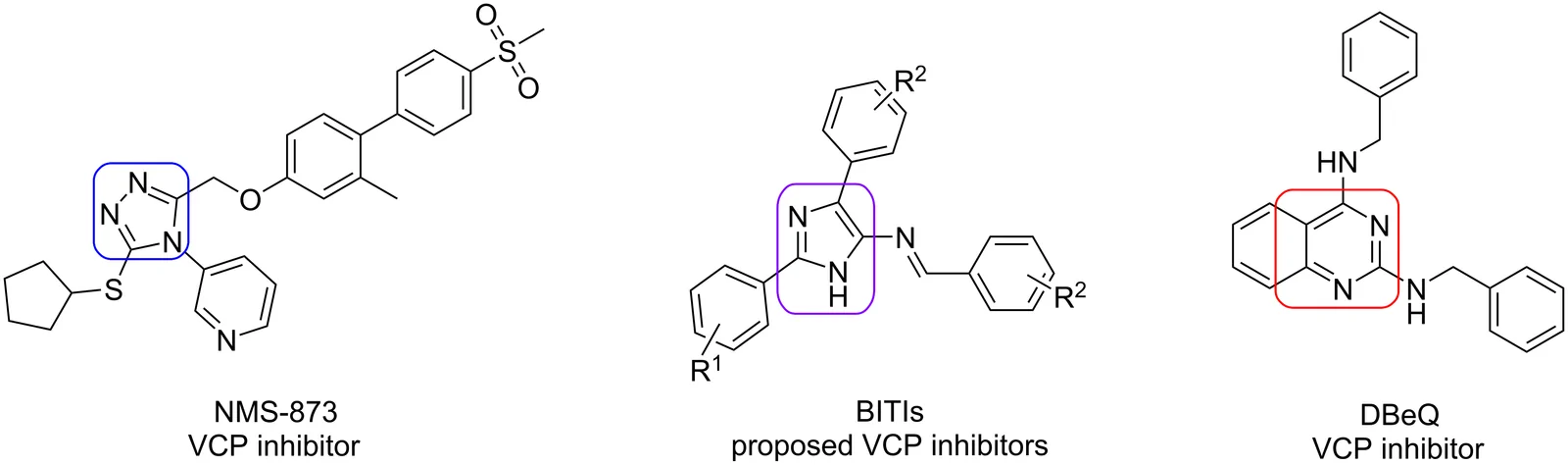
Structures of two known VCP inhibitors, (left) NMS-873 and (right) DBeQ, and (center) the proposed VCP inhibitors, BITIs.

Allosteric binders of VCP have been examined as alternative inhibitors to target its enzymatic activity [10–11,13,16]. NMS-873 is an allosteric binder of VCP, which consists of a triazole core with three substituents attached (Figure 1) [3,13]. However, the synthesis of NMS-873 requires six linear steps [10–11]. Herein, we report the design of VCP inhibitor candidates using scaffold hopping based on previously identified allosteric binders of VCP. Gathering inspiration from the NMS series and the VCP inhibitor *N*^2^,*N*^4^-dibenzylquinazoline-2,4-diamine (DBeQ), we designed a trisubstituted imidazole moiety that could be easily tuned at the 1, 2, and 4 positions (Figure 1). Subsequently, molecular docking was performed to evaluate potential binding within the VCP allosteric site. The top docking pose suggested interactions with Val493, Val497, and Phe618, residues that have been identified as key for allosteric binding (Figure S1, Supporting Information File 1) [16]. The docking results provided preliminary support for the proposed binding hypothesis. These compounds can be synthesized through a microwave-assisted Groebke–Blackburn–Bienaymé (GBB) reaction [17–21]. This multicomponent reaction offers a facile route to synthesize libraries of compounds for rapid biological testing.

Smith et al. recently reported that acyclic amidines are suitable for the GBB reaction [18]. They produced a library of compounds called “benzyl imine tricyclic imidazoles” (BITIs) [18]. Benzamidines were especially promising, showing reactivity with electron-poor and -rich aldehydes [18]. However, the evaluation of substituted benzamidines and their effect on imidazole formation has not been previously reported. Therefore, this study evaluates the effect of benzamidine substitution on the efficiency of the GBB reaction under standard microwave conditions. We aim to identify trends associated with the electronic effects of benzamidines and the most productive substrate combinations.

To further explore the role of amidines in the microwave-assisted GBB reaction, we have employed new benzamidines with electronic and steric differences at the *para*-position in the microwave-assisted GBB reaction. We have also extended our investigation of aromatic aldehydes by investigating disubstituted precursors and by combining the most electron-rich monosubstituted aldehyde with various amidines. Further, compounds were tested for VCP inhibition and cytotoxicity in cancer cells.

## Results and Discussion

### Synthesis of new imidazoles

**Scheme 1:**
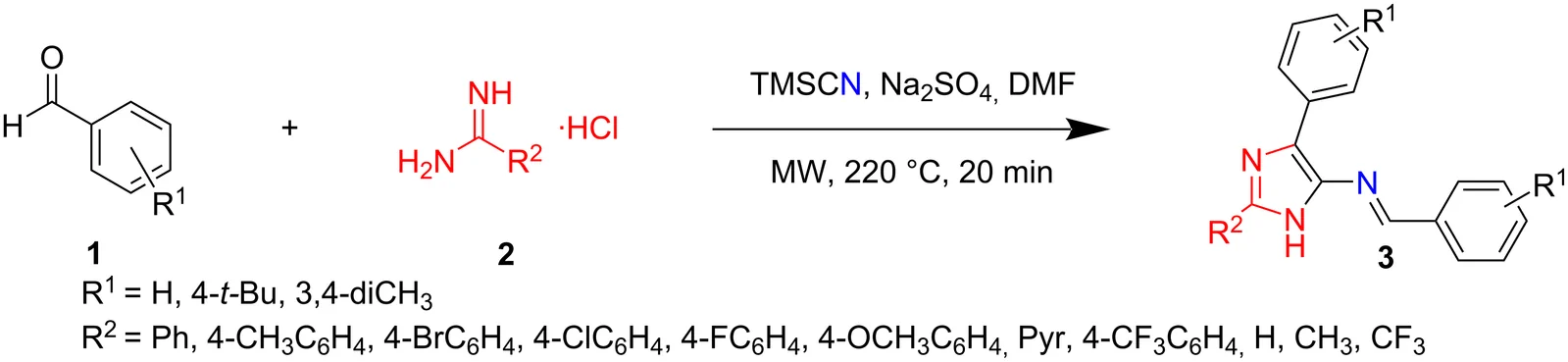
General scheme for the synthesis of imidazoles with various amidines.

**Figure 2:**
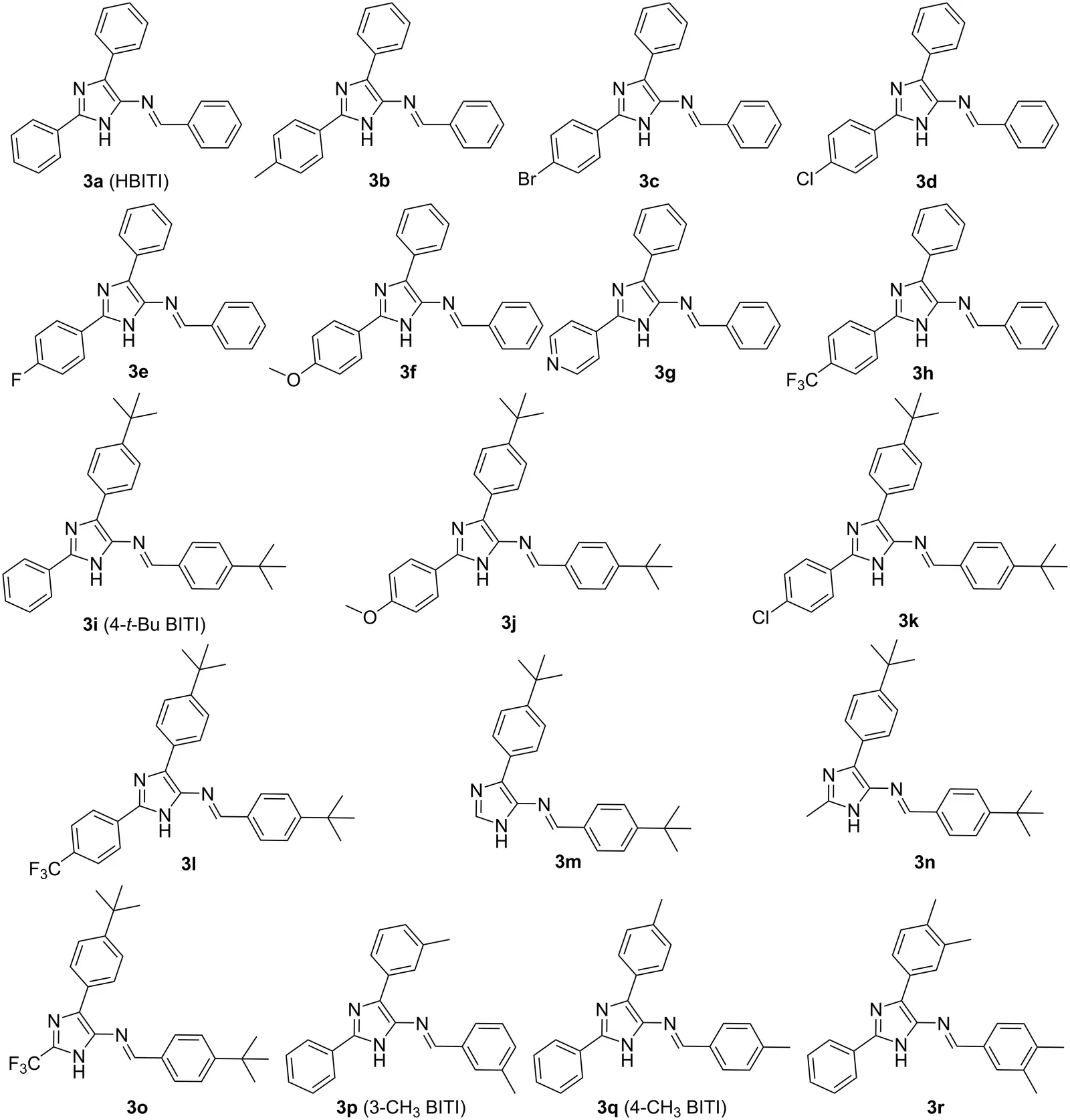
Structures of the proposed imidazoles synthesized via the microwave-assisted GBB reaction.

To directly compare substrate-dependent effects, reactions were performed under standard microwave conditions (220 °C, dimethylformamide (DMF) for 20 min). This approach allowed the assessment of the effect of the benzamidine substitution on the reaction yield. Several substituted benzamidines were used in the synthesis of imidazole compounds via the microwave-assisted GBB reaction (Scheme 1 and Figure 2). 4-Methoxybenzamidine (R^2^ = 4-OMe Ph, **3f**; Table 1, entry 6) afforded a high yield compared to the parent compound, H-BITI (70% **3f** vs 61% **3a**; Table 1, entry 1). Compared to **3f**, 4-methylbenzamidine (**3b**; Table 1, entry 2), 4-bromobenzamidine (**3c**; Table 1, entry 3), 4-chlorobenzamidine (**3d**; Table 1, entry 4), 4-fluorobenzamidine (**3e**; Table 1, entry 5), and 4-amidinopyridine (**3g**; Table 1, entry 7) exhibited lower yields of 37%, 50%, 35%, 43%, and 27%, respectively. Benzamidines with strong electron-withdrawing groups, such as 4-(trifluoromethyl)benzamidine, exhibited the lowest yields (12% **3h**; Table 1, entry 8).

**Table 1:** Yields of the GBB reaction with various amidines (microwave-assisted GBB reaction, 220 °C, *N*,*N*-dimethylformamide (DMF), 20 min).

Entry	Compound	R^1^	R^2^	Yield

1	**3a** (H-BITI)	H	Ph	61% [18]
2	**3b**	H	4-CH_3_C_6_H_4_	37%
3	**3c**	H	4-BrC_6_H_4_	50%
4	**3d**	H	4-ClC_6_H_4_	35%
5	**3e**	H	4-FC_6_H_4_	43%
6	**3f**	H	4-OCH_3_C_6_H_4_	70% [21]
7	**3g**	H	4-Pyr	27%
8	**3h**	H	4-CF_3_C_6_H_4_	12%
9	**3i**	4-*t*-Bu	Ph	80% [18]
10	**3j**	4-*t*-Bu	4-OCH_3_C_6_H_4_	70%
11	**3k**	4-*t*-Bu	4-ClC_6_H_4_	36%
12	**3l**	4-*t*-Bu	4-CF_3_C_6_H_4_	19%
13	**3m**	4-*t*-Bu	H	NR^a^
14	**3n**	4-*t*-Bu	CH_3_	NR^a^
15	**3o**	4-*t*-Bu	CF_3_	NR^a^
16	**3p**	3-CH_3_	Ph	63% [18]
17	**3q**	4-CH_3_	Ph	57% [18]
18	**3r**	3,4-dimethyl	Ph	59%

^a^NR = no detectable product via TLC.

Next, we sought to improve the reaction yield by combining the previously identified highest-yielding aldehyde, 4-*tert*-butylbenzaldehyde, with 4-methoxybenzamidine. The goal of this effort was to identify the most productive substrate combinations and to evaluate the influence of the benzamidine substitution on the GBB reaction. Based on the high reactivity for the individual substrates, we hypothesized that combining the most productive benzaldehyde derivative with the most productive substituted benzamidine would result in a higher yield via additive effects. Surprisingly, this reaction did not have an additive effect, affording a yield of 70% (**3j**; Table 1, entry 10), which is the same yield as the reaction of 4-methoxybenzamidine with benzaldehyde (**3f**; Table 1, entry 6). The reaction of 4-*tert*-butylbenzaldehyde with other benzamidines (**3k** and **3l**; Table 1, entries 11 and 12) also showed improved yields compared to compounds lacking 4-*tert*-butyl substituents (**3d** and **3h**; Table 1; entries 4 and 8). 4-*tert*-Butylbenzaldehyde was also reacted with aliphatic amidines, but no product was detected via thin-layer chromatography (TLC; **3m**–**o**; Table 1; entries 13–15).

Next, we investigated the potential for the additive effect of multiple substituents on the aromatic aldehyde. Because alkyl groups have previously been shown to afford high yields [18], we explored 3,4-dimethylbenzaldehyde. 3,4-Dimethylbenzaldehyde afforded yields comparable to the parent compound, however, the effect was not additive (**3p**–**r**; Table 1; entries 16–18).

### Biological activity

**Figure 3:**
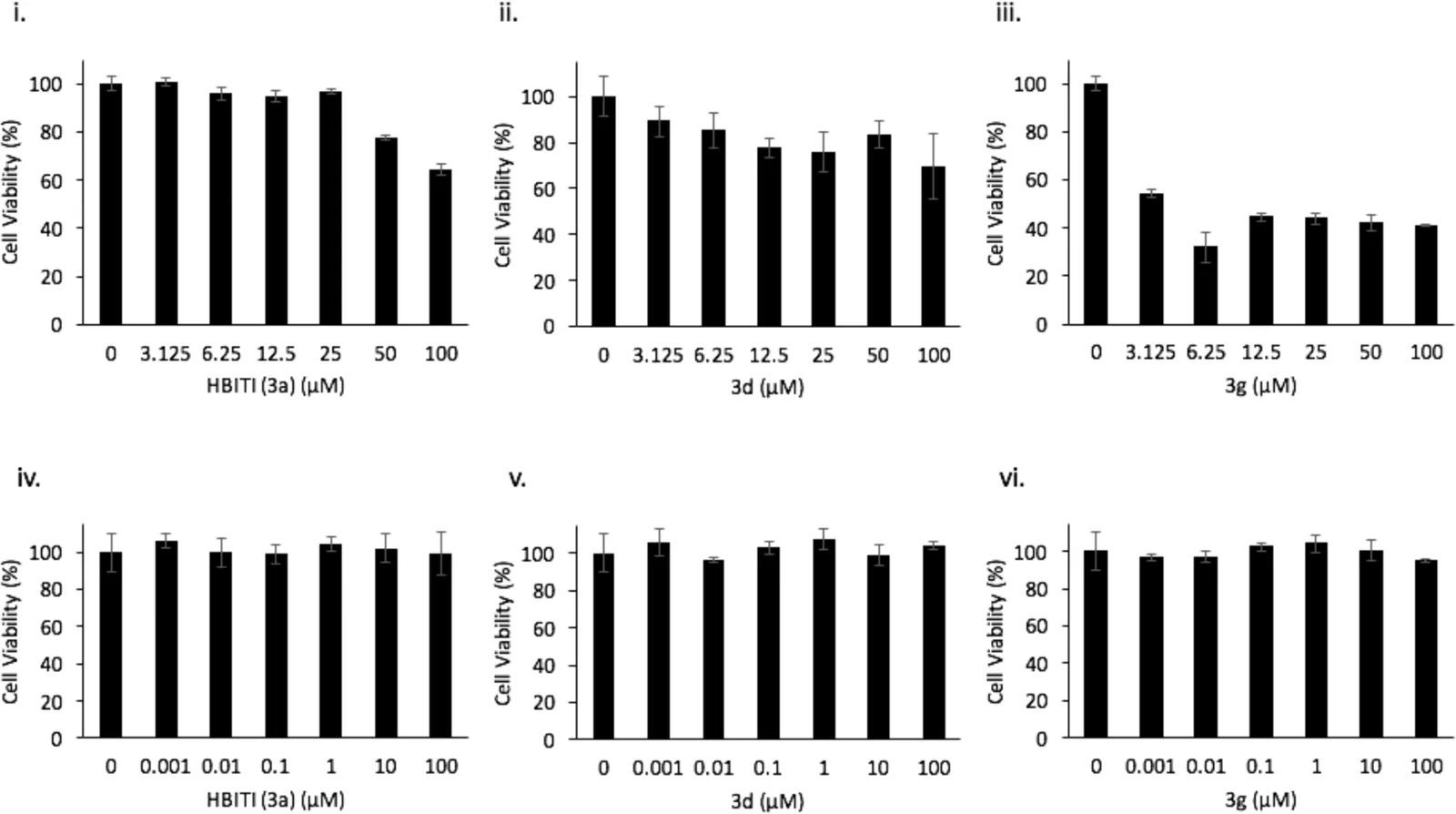
Effect of (i) H-BITI (**3a**), (ii) **3d**, and (iii) **3g** on the cell viability of human breast carcinoma cells (MCF7) after 24 h treatment. Effect of (iv) H-BITI (**3a**), (v) **3d**, and (vi) **3g** on the cell viability of normal human breast cells (MCF10A) after 24 h treatment period.

The biological effects of compounds **1**–**7** were assessed using a malachite green ATPase activity assay, which revealed varying degrees of inhibition of VCP ATPase activity (Figure S2, Supporting Information File 1). This assay measures ATP hydrolysis by detecting the inorganic phosphate released during the reaction. The phosphate forms a complex with the dye malachite green and molybdate to produce a green color that can be measured spectrophotometrically [10]. All tested compounds inhibited ATPase activity to varying degrees. The amidinopyridine derivative **3g** exhibited the strongest inhibition, prompting further investigation into the biological effects of this compound and other selected compounds. Compound **3d** was selected owing to the known ability of chlorination to enhance potency and improve stability and hydrophobicity in drug design [22]. A dose-dependent cytotoxicity in human breast carcinoma cells (MCF7) was observed for the parent compound **3a**, **3d**, and **3g** with **3g** showing significantly higher cytotoxicity (IC_50_ ≈ 3.25 μM) than **3a** and **3d** (IC_50_ > 100 μM; Figure 3i–iii). In contrast, compounds **3a**, **3d**, and **3g** were not cytotoxic to normal human breast cells (MCF10a; Figure 3iv–vi). Although **3g** exhibits modest inhibition of VCP activity in vitro, its enhanced cellular toxicity may reflect allosteric binding modes or additional cellular effects downstream of VCP inhibition. Further studies are required to determine the precise mechanism of action.

## Conclusion

This study of substituted benzamidines and disubstituted aldehydes in the GBB reaction provides additional insights into the role of the electronic properties of amidines in the GBB reaction. In our earlier study, we showed that the substituents on aromatic aldehydes can affect the reaction yield, with electron-donating groups achieving a high yield [18]. This study shows that substituents on the benzamidine can also tune the reaction. 4-Methoxybenzamidine afforded the highest yield, which suggests that electron donation via resonance by benzamidine also promotes this reaction. This is supported by the comparison of the GBB reaction of 4-methylbenzamidine and 4-methoxybenzamidine, which show yields of 37% and 70%, respectively (**3b** vs **3f**; Table 1). These differences suggest that the resonance donation of the methoxy group is more effective than the inductive donation of the methyl group. This is likely because the methoxy group increases the nucleophilicity of the amine during imine formation in the initial step of the GBB reaction. The non-additive effect of the disubstituted substrates further supports the need for resonance donation to significantly improve the yields. Further exploration of benzamidines with various alkyl groups and additional electron-donating substituents is required for confirmation. However, these efforts may necessitate the development of new methods for synthesizing amidines. Additionally, microwave irradiation can interfere with the subtle electronic effects of this reaction. Future studies will involve exploring these reactions under non-microwave conditions to further understand these trends.

In addition, the selective biological effect of **3a** and the amidinopyridine derivative **3g** on breast cancer cells indicates that these imidazole derivatives warrant further investigation as potential therapeutic agents targeting VCP-associated pathways in cancer. Future studies will involve designing derivatives of **3g** to further understand its biological effects and protein targets. In brief, this study demonstrated that the expansion of the GBB reaction to acyclic amidines, specifically substituted benzamidine, enables the rapid formation of diverse imidazole-based compounds with biological selectivity.

## Experimental

### General procedure for the synthesis of benzyl imine tricyclic imidazoles (BITIs)

In a reaction vessel, benzamidine hydrochloric acid (0.05 g, 1 equiv, 0.5 mmol) and sodium sulfate (0.3 g, 4 equiv, 2 mmol) were suspended in anhydrous DMF (2.5 mL). Benzaldehyde (0.2 g, 0.4 mL, 4 equiv, 2 mmol) and trimethylsilanecarbonitrile (0.1 g, 0.1 mL, 2 equiv, 1 mmol) were added, and the solution was irradiated in a microwave at 220 °C for 10 min. The reaction mixture was extracted with water and ethyl acetate to remove DMF. The solvent was removed, and the crude product was purified by column chromatography (Hex/EtOAc).

### VCP ATPase activity assay

VCP ATPase activity assays were performed in a reaction buffer containing 50 mM Tris-HCl (pH 7.5; Invitrogen), 0.2 mg/mL BSA (Sigma), 10 mM MgCl_2_ (Hampton Research), and 2 mM dithiothreitol (DTT; Sigma). Assays were performed in clear 96-well plates. First, 150 nM VCP was preincubated with each compound (100 µM) in the reaction buffer for 20 min at 37 °C. Then, the reaction was initiated by addition of 75 µM ATP to make a final volume of 80 µL per well, and the reaction was allowed to proceed for 60 min at 37 °C. A malachite green phosphate assay kit (Sigma) was used according to the manufacturer’s instructions to determine the levels of free phosphate. A standard phosphate curve was prepared in reaction buffer to determine the phosphate concentration. The absorbance was measured at 620 nm using a Spectramax iD5e microplate reader. All reactions were performed in triplicate, with vehicle controls included at the same final concentration for compound treatments. Data are expressed as mean ± standard deviation (SD).

### Cell viability assay

MCF7 cells were maintained in Dulbecco’s modified Eagle’s medium (Gibco) supplemented with 10% FBS (Gibco) and 1% penicillin/streptomycin (Gibco) in a 5% CO_2_ humidified incubator at 37 °C. The cell viability of MCF7 cells was determined using the CyQUANT 3-(4,5-dimethylthiazol-2-yl)-2,5-diphenyltetrazolium bromide (MTT) cell viability assay kit (Invitrogen) according to the manufacturer’s instructions. MCF7 cells were seeded at a density of approximately 10,000 cells/well in 96-well clear-bottom plates (Celltreat) overnight. The culture medium was removed, and various concentrations (0–100 µM) of selected compounds diluted in culture medium (100 µL/well) were added and incubated at 37 °C in 5% CO_2_ for 24 h. The medium was removed, and 100 µL of fresh culture medium was added to each well. Next, 10 µL of a 12 mM MTT stock solution diluted in phosphate-buffered saline was added to each well. The plate was incubated for 4 h, followed by the addition of 100 µL of a sodium dodecyl sulfate (SDS) HCl solution. The plate was incubated at 37 °C in 5% CO₂ for 18 h. After incubation, the absorbance was measured at 570 nm using a BMG Labtech FLUOstar Omega plate reader. All treatments were performed in triplicate, and vehicle-treated cells served as controls.

## Supporting Information

File 1 Docking results, VCP ATPase activity from a malachite green enzymatic assay, synthetic procedures for BITIs, and nuclear magnetic resonance spectroscopy data for **3b**–**h**, **3j**–**l**, and **3r**.

## Data Availability

All data that supports the findings of this study is available in the published article and/or the supporting information of this article.
