# Supplementary material for: Expanded use of benzamidines in the Groebke–Blackburn–Bienaymé synthesis of imidazoles and their biological evaluation
Source: Beilstein J Org Chem. 2026 Sep 30;22:1344–50. doi: 10.3762/bjoc.22.108 (PMC13635903; doi:10.3762/bjoc.22.108)
Supplement: Supplementary file 1 [file Beilstein_J_Org_Chem-22-1344-s001.pdf]

BEILSTEIN JOURNAL OF ORGANIC CHEMISTRY

Supporting Information

for

Expanded use of benzamidines in the Groebke–Blackburn–Bienaymé synthesis of imidazoles and their biological evaluation

Kamryn Walker, Sydney Rivas, Erik Tillman-Smith, Kayla McLeod, Jeanee Bullock and Jacqueline Smith

Beilstein J. Org. Chem. **2026,** *22,* 1344–1350. doi:10.3762/bjoc.22.108

Docking results, VCP ATPase activity from a malachite green enzymatic assay, synthetic procedures for BITIs, and nuclear magnetic resonance spectroscopy data for 3b–h, 3j–l, and 3r

License and Terms: This is a supporting information file under the terms of the Creative Commons Attribution License (https://creativecommons.org/licenses/by/4.0). Please note that the reuse, redistribution and reproduction in particular requires that the author(s) and source are credited and that individual graphics may be subject to special legal provisions.
The license is subject to the *Beilstein Journal of Organic Chemistry* terms and conditions: (https://www.beilstein-journals.org/bjoc/terms)

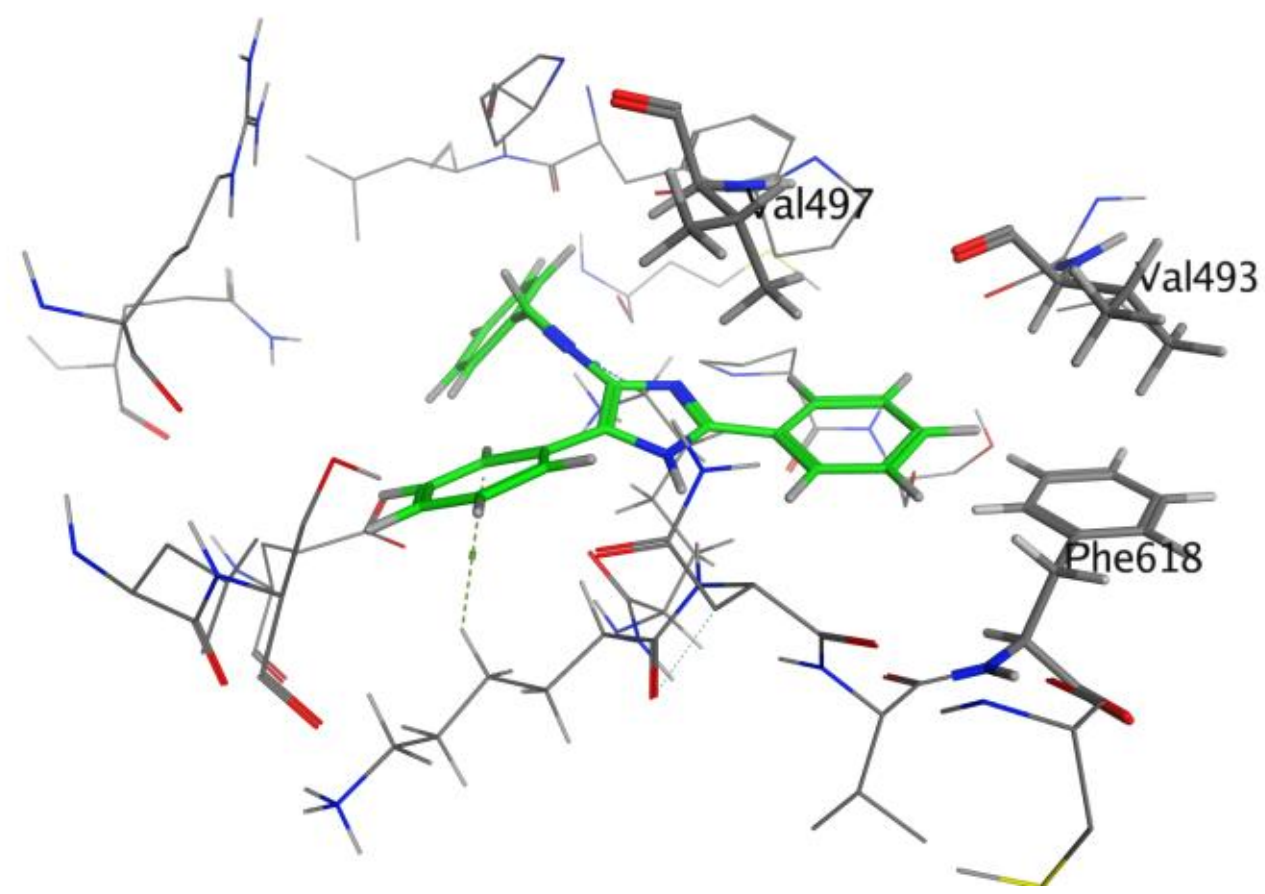

Figure S1: Molecular docking was performed using the cryo-EM structure of VCP (PDB ID: 5FTJ) in MOE. The top-ranked docking pose suggests that compound **1** (HBITI) occupies the reported allosteric binding and engages residues Val493, Val 497, Phe618 which are key residues for allosteric binging of VCP inhibitors. Protein residues are shown as sticks and the ligand is shown in green.

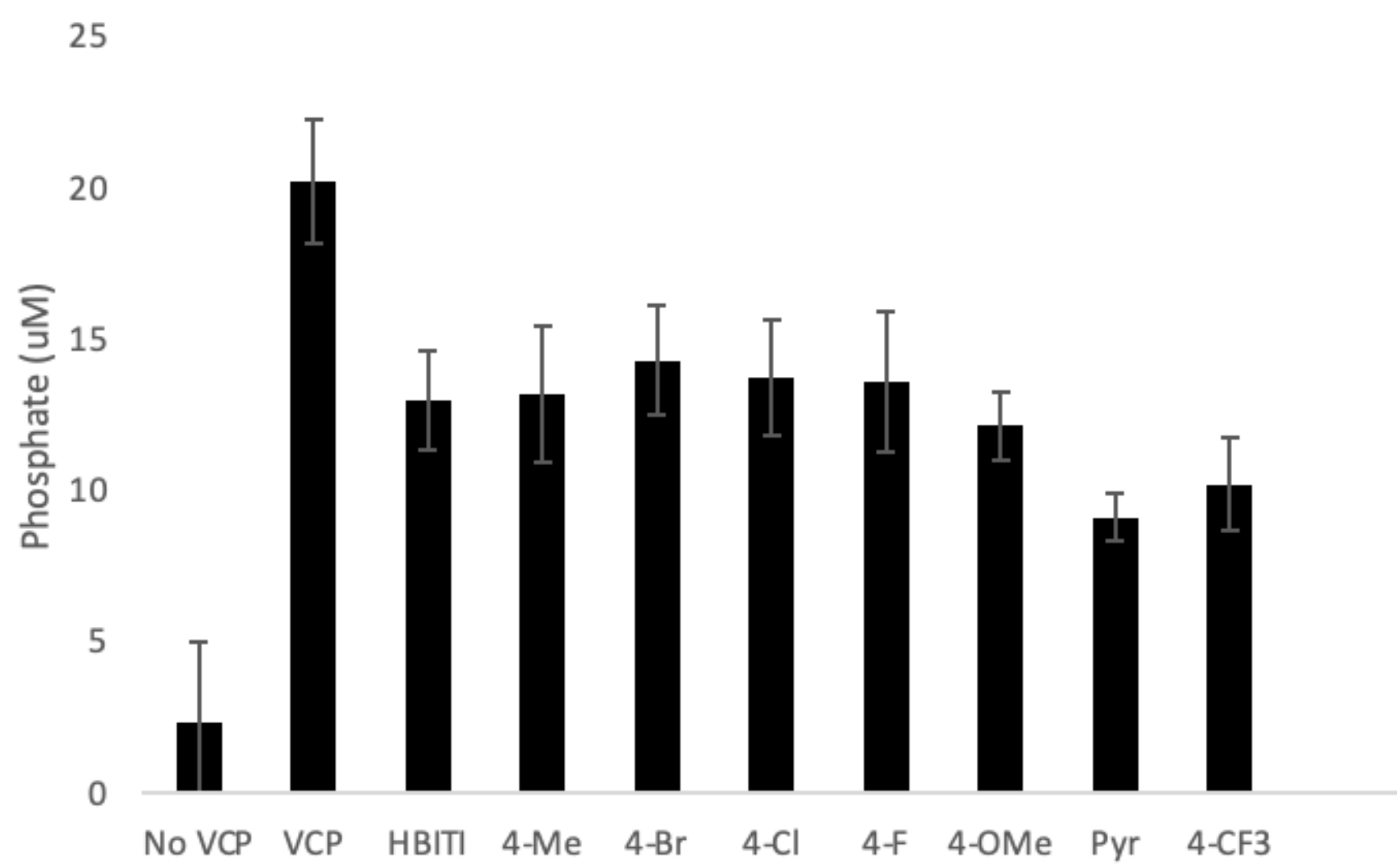

Figure S2: Effect of compounds (100 micromolar) on VCP ATPase activity using a malachite green enzymatic assay.

General

All reagents and solvents were purchased from commercial suppliers and used as received unless noted otherwise. Flash column chromatography separations were done on a Biotage Select purification system, monitoring at 254 and 280 nm. NMR spectra were recorded on a Bruker Advance spectrometer at 25 °C, operating at 500 MHz for ^{1}H and 126 MHz for ^{13}C NMR. ^{1}H NMR and ^{13}C NMR data are reported as follows: chemical shift in ppm (δ), multiplicity (s = singlet, d = doublet, t = triplet, m = multiplet). Coupling constants are measured in hertz (Hz). The chemical shifts are expressed in ppm downfield from TMS as an internal standard. High-resolution mass spectra (HRMS) were recorded using electrospray ionization (ESI) in positive mode on a JEOL AccuTOF-CS spectrometer, reported as *m*/*z* values for $[M + H]^+$. Reactions were monitored by thin-layer chromatography (TLC) on silica gel aluminum slides. All microwave experiments were conducted in an Anton-Paar Monowave 450. The reactions were carried out in a 30 mL glass vial with a septum. The reaction was irradiated at the prescribed temperature for the stipulated time and then cooled to 55 °C.

General procedure for synthesis of 2,4-benzyl-5-imino tricyclic imidazoles (BITIs)

In the reaction vessel, benzamidine hydrochloric acid (0.05 g, 1 equiv, 0.5 mmol) and sodium sulfate (0.3 g, 4 equiv, 2 mmol) were suspended in anhydrous DMF (2.5 mL). Benzaldehyde (0.4 g, 0.4 mL, 8 equiv, 4 mmol), and trimethylsilanecarbonitrile (0.1 g, 0.1 mL, 2 equiv, 1 mmol) were added and the solution was microwave irradiated at 220 °C for 20 min. The reaction mixture was extracted with water and ethyl acetate to remove the DMF. The solvent was removed and the crude product was purified by column chromatography (Hex/EtOAc).

(E)-N-Benzylidene-4-phenyl-2-(p-tolyl)-1H-imidazol-5-amine (3b) A light yellow powder was obtained (52 mg, 37%). R_f 0.88 (silica gel, 5:1 hexane/EtOAc) ^{1}H NMR (500 MHz, $CDCl_3$) δ 9.37 (s, 1H), 9.32 (s, 1H), 7.96-7.99 (m, 4H), 7.80 (d, *J* = 8.2 Hz, 2H), 7.42-7.48 (m, 5H), 7.31 (t, *J* = 3.2 Hz, 1H), 7.27 (d, *J* = 7.9 Hz, 2H), 2.40 (s, 3H). ^{13}C NMR (126 MHz, $CDCl_3$) δ 156.5, 144.6, 19.3, 137.1, 130.5, 129.7, 128.7, 128.6, 127.2, 127.0, 126.4, 125.2, 21.4 $[M+H]^+$(ESI): calc'd for $C_{23}H_{19}N_3$, 338.426; found 338.164

(E)-N-Benzylidene-2-(4-bromophenyl)-4-phenyl-1H-imidazol-5-amine (3c) A dark, brown powder was obtained (81 mg, 50%). R_f 0.8 (silica gel, 5:1 hexane/EtOAc) ^{1}H NMR (500 MHz, $CDCl_3$) δ 9.39 (s, 1H), 9.31 (s, 1H), 7.96-7.98 (m, 4H), 7.79 (d, *J* = 8.6 Hz, 2H), 7.60 (d, *J* = 8.6 Hz, 2H), 7.44-7.50 (m, 5H), 7.33 (t, *J* = 7.4 Hz, 1H). ^{13}C NMR (126 MHz, $CDCl_3$) δ 157.0, 143.2, 137.0, 132.2, 130.7, 128.9, 128.7, 128.6, 127.4, 126.7, 126.5, 123.3 $[M+H]^+$(ESI): calc'd for, $C_{22}H_{16}N_3Br_,$ 402.295, 404.051; found 402.059, 404.060

(E)-N-Benzylidene-2-(4-chlorophenyl)-4-phenyl-1H-imidazol-5-amine (3d) A light yellow powder was obtained (50 mg, 35%). R_f 0.55 (silica gel, 5:1 hexane/EtOAc) ^{1}H NMR (500 MHz, $CDCl_3$) δ 9.41 (s, 1H), 9.31 (s, 1H), 7.96-7.98 (m, 4H), 7.85 (d, *J* = 8.6 Hz, 2H), 7.47 (m, 8H), 7.33 (t, *J* = 7.4 Hz, 1H). ^{13}C NMR (126 MHz, $CDCl_3$) δ 156.9, 145.5, 143.2, 137.0, 135.0, 130.6, 129.2, 128.8, 128.7, 128.6, 128.2, 126.5 $[M+H]^+$(ESI): calc'd for, $C_{22}H_{18}N_3$ $Cl_,$ 358.100; 360.001 found 358.111; 360.111

(E)-N-Benzylidene-2-(4-fluorophenyl)-4-phenyl-1H-imidazol-5-amine (3e) A tan, brown powder was obtained (57 mg, 43%). R_f 0.63 (silica gel, 5:1 hexane/EtOAc) ^{1}H NMR (500 MHz, $CDCl_3$) δ 9.43 (s, 1H), 9.31 (s, 1H), 7.95-7.98 (m, 4H), 7.86-7.89 (m, 2H), 7.43-7.49 (m, 5H), 7.32 (t, J = 7.4 Hz, 1H), 7.15 (t, J = 8.6 Hz, 2H). ^{13}C NMR (126 MHz, $CDCl_3$) δ 116.0, 116.2, 124.9, 126.1, 126.2, 126.4, 127.2, 127.3, 128.6, 128.7, 128.8, 129.6, 130.6, 137.0, 143.6, 145.4, 156.8, 162.3, 164.3 $[M+H]^+$(ESI): calc'd for, $C_{22}H_{16}N_3F$, 342.389 ; found 342.141

(E)-N-Benzylidene-2-(4-methoxyphenyl)-4-phenyl-1H-imidazol-5-amine (3f) A tan, brown powder was obtained (80 mg, 70%). R_f 0.61 (silica gel, 5:1 hexanes/EtOAc) ^{1}1H NMR (500 MHz, CDCl3) δ 9.87 (s, 1H), 9.30 (s, 1H), 8.01 (d, J = 5.5 Hz, 2H), 7.95 (d, J = 6.7 Hz, 2H), 7.86 (d, J = 8.8 Hz, 2H), 7.41-7.46 (m, 5H), 7.27-7.30 (m, 2H), 6.95 (d, J = 8.8 Hz, 2H), 3.83 (s, 3H) ^{13}C NMR (126 MHz, $CDCl_3$) δ 162.7, 160.4, 156.3, 144.8, 137.3, 130.4, 128.7, 128.6, 126.5, 122.7, 114.3, 55.4 $[M+H]^+$(ESI): calc'd for $C_{23}H_{19}N_3O$, 354.425; found 354.161 [1]

(E)-N-Benzylidene-4-phenyl-2-(pyridin-4-yl)-1H-imidazol-5-amine (3g) A tan, brown powder was obtained (70 mg, 27%). R_f 0.57 (silica gel, 6:1 hexane/EtOAc) ^{1}H NMR (500 MHz, $CDCl_3$) δ 9.74 (s, 1H), 9.35 (s, 1H), 8.72–8.73 (m, 2H), 8.00-8.01 (m, 4H), 7.81-7.82 (m, 2H), 7.47-7.53 (m, 6H), 7.37 (t, J = 7.4 Hz, 1H) ^{13}C NMR (126 MHz, $CDCl_3$) δ 119.1, 119.2, 126.8, 127.3, 127.7, 128.7, 128.8, 130.9, 136.9, 137.0, 141.4, 150.3 $[M+H]^+$(ESI): calc'd for $C_{21}H_{16}N_4$, 324.38; found 325.146

(E)-N-Benzylidene-4-phenyl-2-(4-(trifluoromethyl)phenyl)-1H-imidazol-5-amine (3h) A light yellow powder was obtained (20 mg, 12%). R_f 0.67 (silica gel, 5:1 hexane/EtOAc) ^{1}H NMR (500 MHz, $CDCl_3$) δ 9.55 (s, 1H), 9.27 (s, 1H), 7.98 (d, J = 8.1 Hz, 2H), 7.91-7.94 (m,4H), 7.66 (d, J = 8.2 Hz, 2H), 7.39-7.44 (m, 5H), 7.28 (t, J = 7.4 Hz, 2H). ^{13}C NMR (126 MHz, $CDCl_3$) δ 125.4, 126.0, 126.5, 127.6, 128.6, 128.8, 128.9, 130.7, 142.6, 145.8, 147.3 $[M+H]^+$(ESI): calc'd for, $C_{23}H_{18}N_3 F_3$, 392.130; found 392.138

4-(E)-N-(4-(tert-Butyl)benzylidene)-4-(4-(tert-butyl)phenyl)-2-(4-methoxyphenyl)-1H-imidazol-5-amine (3j) A dark brown powder was obtained (132 mg, 70%). R_f 0.56 (silica gel, 5:1 hexanes/EtOAc) ^{1}H NMR (500 MHz, $CDCl_3$) δ 9.29 (s, 1H), 9.19 (s, 1H), 7.92 (dd, J = 4.9, 8,3 Hz, 4H), 7.85 (d, J = 8.8 Hz, 2H), 7.49 (dd, J = 5.6, 8.3 Hz, 4H), 7.00 (d, J = 8.8 Hz, 2H), 3.87 (s, 3H), 1.37 (s, 9H), 1.36 (s, 9H) ^{13}C NMR (126 MHz, $CDCl_3$) δ 31.2, 31.3, 34.6, 34.9, 55.4, 114.4, 122.7, 124.2, 125.5, 125.7, 126.0, 126.7, 128.4, 134.4, 144.1, 145.2, 150.1, 153.7, 155.9, 160.4 $[M+H]^+$(ESI): calc'd for $C_{31}H_{35}N_3O$, 466.641; found 466.287

(E)-1-(4-(tert-Butyl)phenyl)-N-(4-(4-(tert-butyl)phenyl)-2-(4-chlorophenyl)-1H-imidazol-5-yl)methanimine (3k) A yellow, crystalline solid was obtained (86 mg, 36%). R_f 0.35 (silica gel, 9:1 hexanes/EtOAc). ^{1}H NMR (500 MHz, $CDCl_3$) δ 9.20 (br, 1H), 7.92 (d, J = 8.1 Hz, 2H), 7.84 (d, J = 8.1 Hz, 2H), 7.77 (d, J = 8.2 Hz, 2H), 7.43-7.46 (m, 4H), 7.30 (d, J = 7.8 Hz, 2H), 1.34 (s, 18H). ^{13}C NMR (126 MHz, $CDCl_3$) δ 31.22, 31.27, 34.61, 34.93, 125.5, 125.6, 126.5, 126.7, 128.4, 128.9, 134.5, 134.6, 143.2, 150.1, 153.9 [M+H]+(ESI): calc'd for $C_{30}H_{32}ClN_3$, 470.228, 472.232; found 470.235, 472.238

(E)-1-(4-(tert-Butyl)phenyl)-N-(4-(4-(tert-butyl)phenyl)-2-(4-(trifluoromethyl)phenyl)-1H-imidazol-5-yl)methanimine (3l) A yellow-green, crystalline solid was obtained (41 mg, 19%). R_f 0.42 (silica gel, 9:1 hexanes/EtOAc). ^{1}H NMR (500 MHz, $CDCl_3$) δ 9.24 (s, 1H), 8.05 (d, J = 8.0 Hz, 2H), 7.94 (d, J = 8.2 Hz, 2H), 7.89 (d, J = 8.1 Hz, 2H), 7.62 (d, J = 8.1 Hz, 2H), 7.45-7.49 (m, 4H), 1.36 (s, 9H), 1.35 (s, 9H) ^{13}C NMR (126 MHz, $CDCl_3$) δ 31.30, 34.67, 3.98, 125.5, 125.6, 125.8, 126.5, 128.5, 133.3, 134.5, 142.6, 150.4, 154.0, 156.4 [M+H]+(ESI): calc'd for $C_{31}H_{32}F_3N_3$, 504.255; found 504.263

(E)-1-(3,4-Dimethylphenyl)-N-(4-(3,4-dimethylphenyl)-2-phenyl-1H-imidazol-5-yl)methanimine (3r) A dark brown powder was obtained (44 mg, 59%). R_f 0.56 (silica gel, 5:1 hexanes/EtOAc) ^{1}H NMR (500 MHz, $CDCl_3$) δ 10.35 (s, 1H), 9.07 (s, 1H), 7.68 (d, J = 7.4 Hz, 3H), 7.58-7.59 (m, 1H), 7.44-7.48 (m, 2H), 7.10-7.17 (m, 4H), 7.01-7.03 (m, 2H), 2.14 (s, 12H). ^{13}C NMR (126 MHz, $CDCl_3$) δ 19.62, 19.85, 19.99, 20.02, 124.1, 125.5, 126.3, 127.5, 128.0, 129.6, 129.9, 135.1, 135.6, 136.7, 139.5, 144.3, 145.2, 156.2, 162.8 $[M+H]^+$(ESI): calc'd for $C_{26}H_{25}N_3$, 380.507 ; found 380.211

References

1. Bell, C. E.; Shaw, A. Y.; De Moliner, F.; Hulme, C. *Tetrahedron* **2014**, *70*, 54–59.

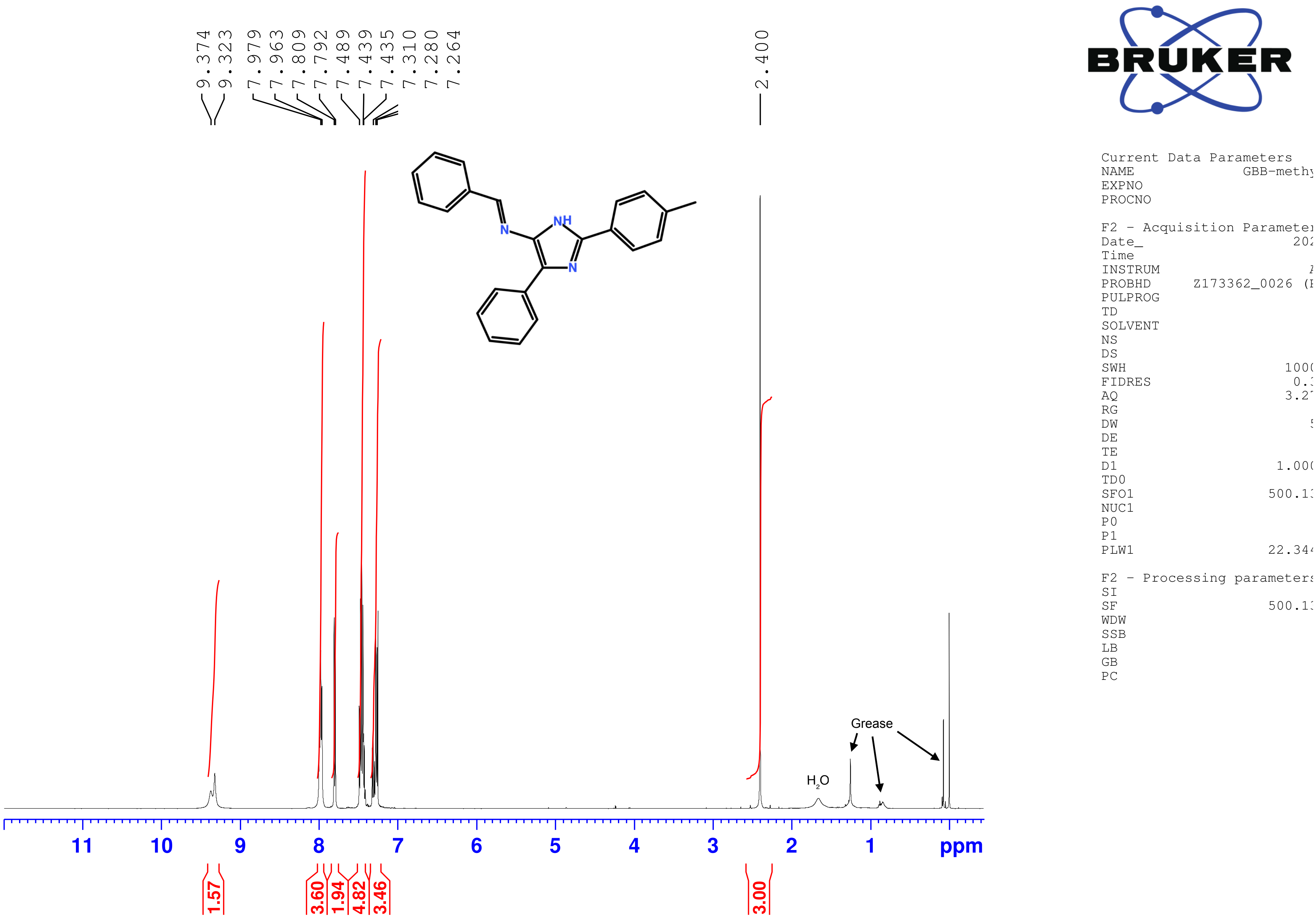
9.374
9.323
7.979
7.963
7.809
7.792
7.489
7.439
7.435
7.310
7.280
7.264
2.400
NH
N
N
H2O
Grease
11 10 9 8 7 6 5 4 3 2 1 ppm
1.57
3.60
1.94
4.82
3.46
3.00
BRUKER
Current Data Parameters
NAME GBB-methy
EXPNO
PROCNO
F2 - Acquisition Paramete
Date_ 202
Time
INSTRUM
PROBHD Z173362_0026 (
PULPROG
TD
SOLVENT
NS
DS
SWH 100
FIDRES 0.
AQ 3.2
RG
DW
DE
TE
D1 1.00
TD0
SFO1 500.1
NUC1
P0
P1
PLW1 22.34
F2 - Processing parameter
SI
SF 500.1
WDW
SSB
LB
GB
PC

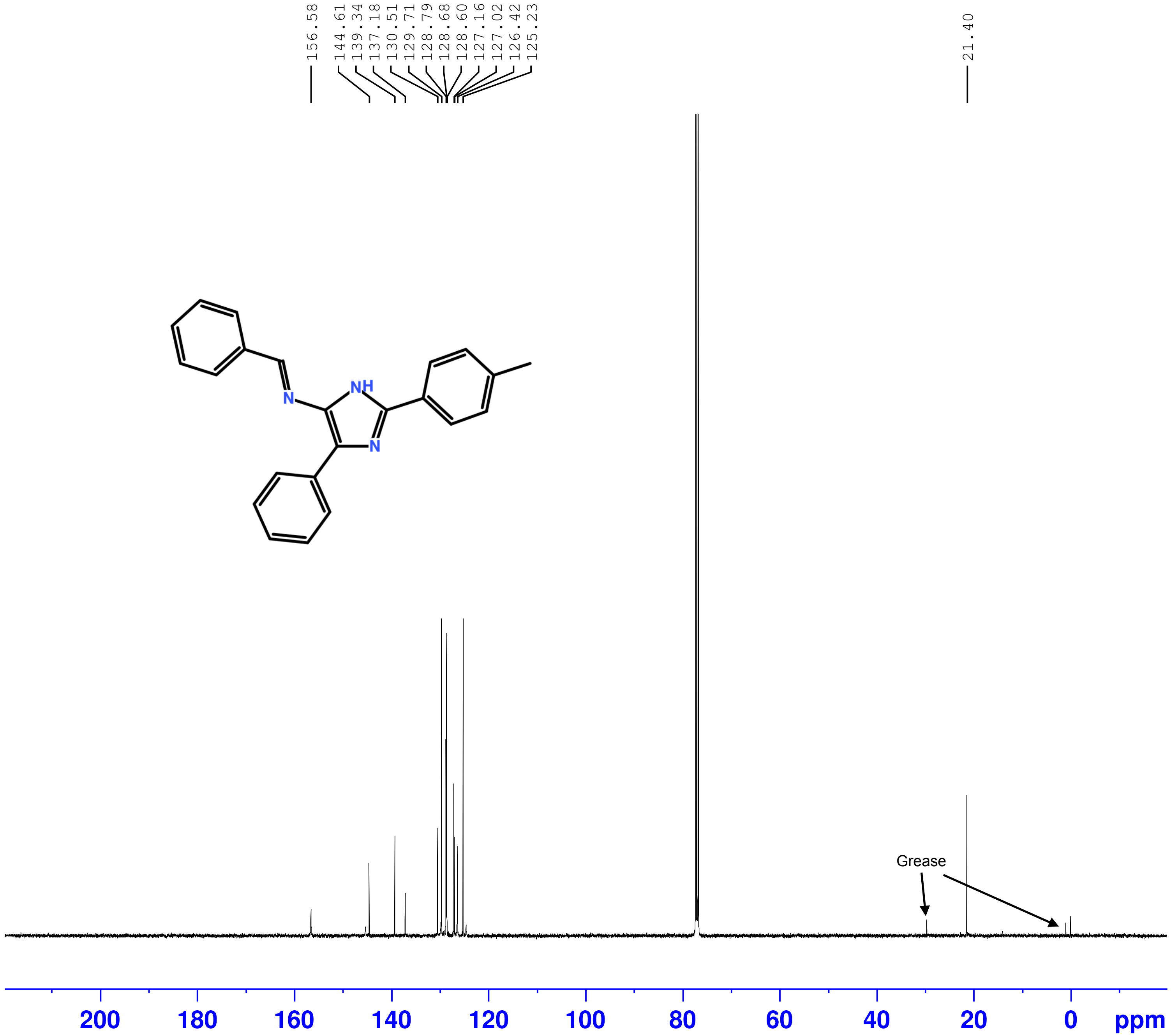

Current Data Parameters	
NAME	GBB-methyl-BAM
EXPNO	2
PROCNO	1
F2 - Acquisition Parameters	
Date_	20250508
Time	1.07
INSTRUM	Avance
PROBHD	Z173362_0026 (PI HR-
PULPROG	zgpg30
TD	65536
SOLVENT	CDCl3
NS	5120
DS	4
SWH	30120.482
FIDRES	0.919204
AQ	1.0878976
RG	101
DW	16.600
DE	6.50
TE	298.0
D1	2.00000000
D11	0.03000000
TD0	1
SFO1	125.7703643
NUC1	13C
P0	3.33
P1	10.00
PLW1	81.01100000
SFO2	500.1320005
NUC2	1H
CPDPRG2	waltz65
PCPD2	80.00
PLW2	22.34400000
PLW12	0.34912000
PLW13	0.17561000
F2 - Processing parameters	
SI	32768
SF	125.7577907
WDW	EM
SSB	0
LB	1.00
GB	0
PC	1.40

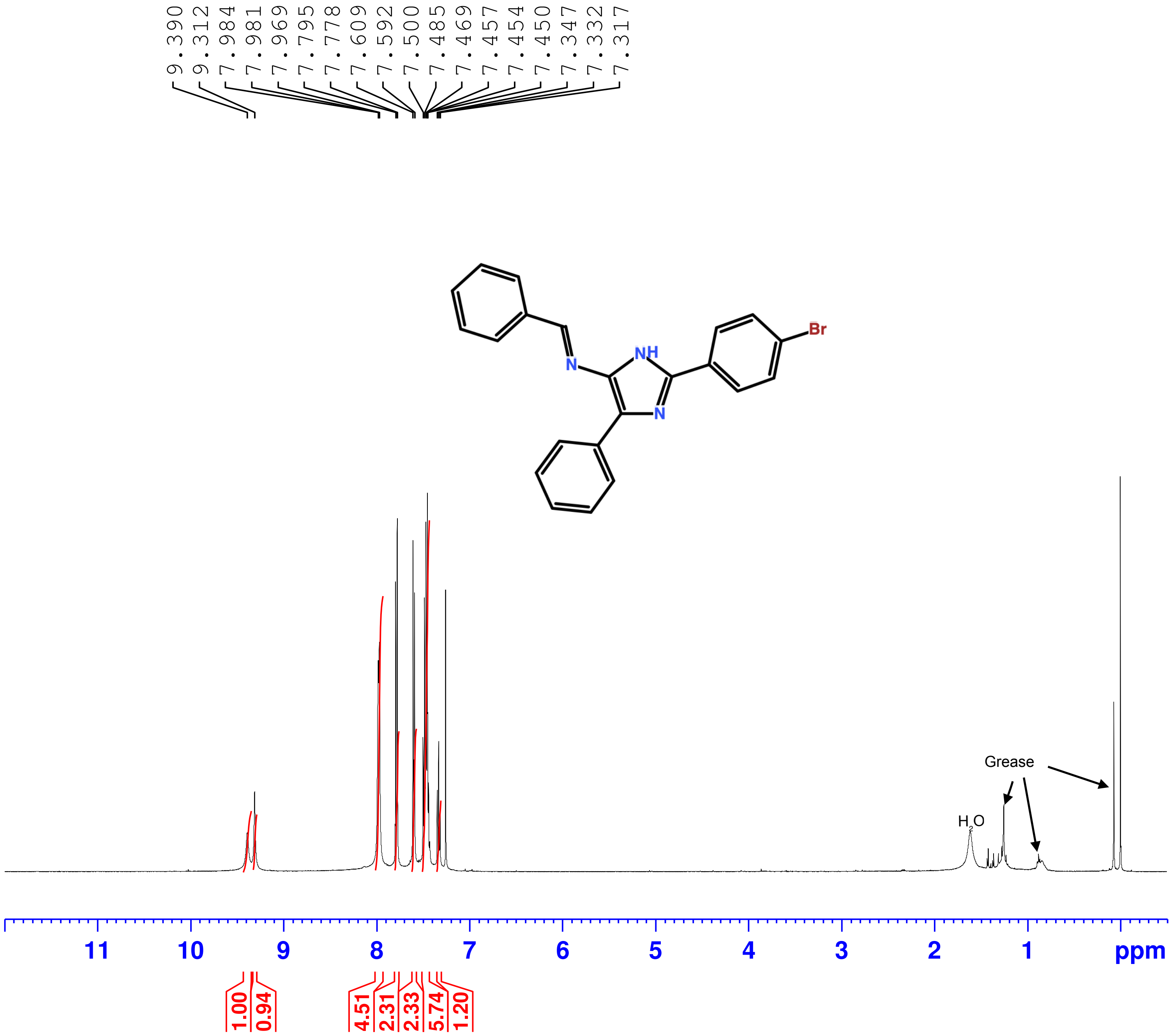

Current Data Parameters
NAME bromo-dry
EXPNO 2
PROCNO 1

F2 - Acquisition Parameters
Date_ 20250520
Time 13.15
INSTRUM Avance
PROBHD Z173362_0026 (PI HR-
PULPROG zg30
TD 65536
SOLVENT CDCl3
NS 16
DS 2
SWH 10000.000
FIDRES 0.305176
AQ 3.2768000
RG 101
DW 50.000
DE 10.79
TE 298.0
D1 1.00000000
TD0 1
SFO1 500.1330883
NUC1 1H
P0 3.33
P1 10.00
PLW1 22.34400000

F2 - Processing parameters
SI 65536
SF 500.1300137
WDW EM
SSB 0
LB 0.30
GB 0
PC 1.00

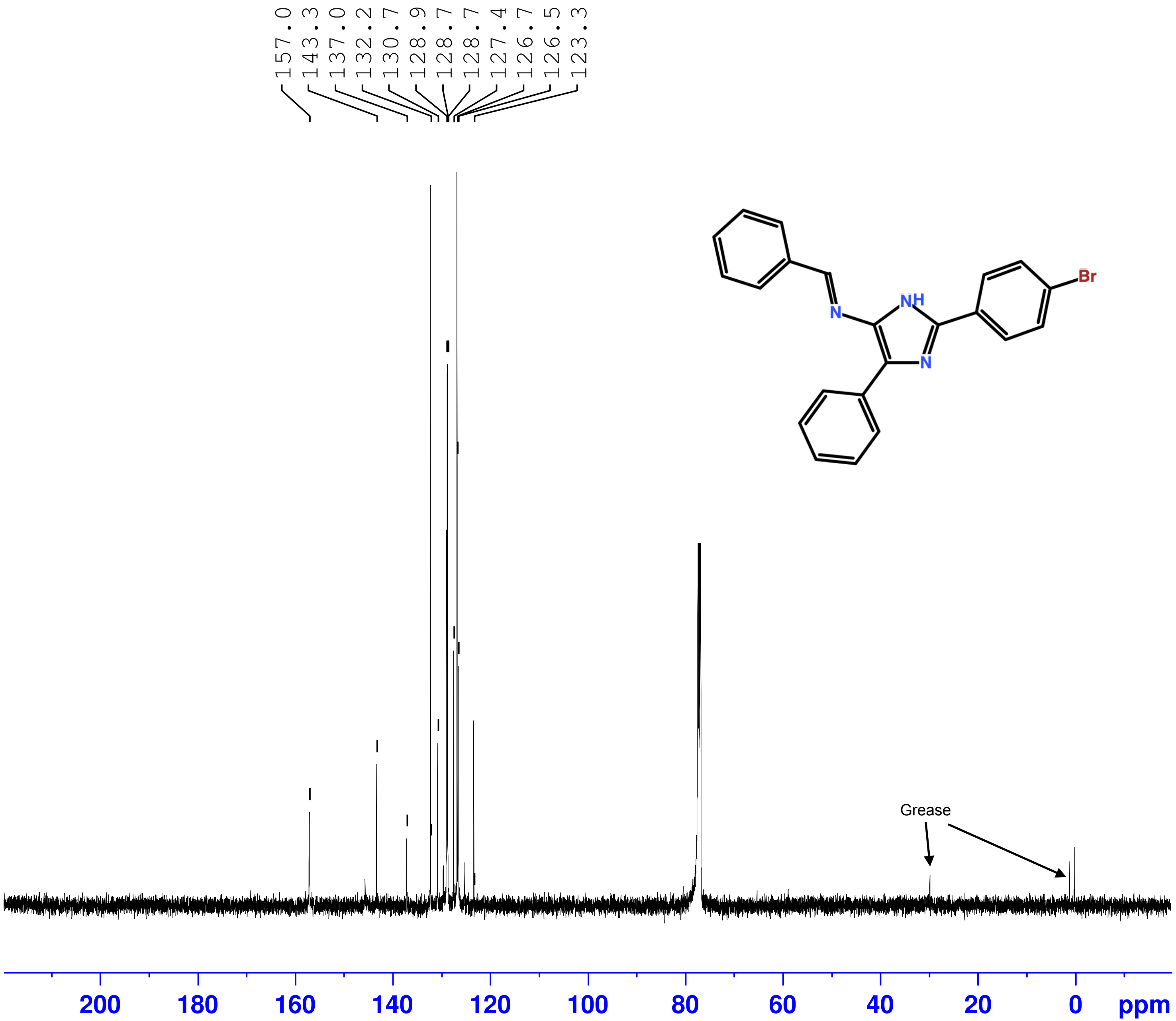

Current Data Parameters

NAME	bromo-carbon-dry
EXPNO	1
PROCNO	1

F2 - Acquisition Parameters

Date_	20250520
Time	18.20
INSTRUM	Avance
PROBHD	Z173362_0026 (PI HR-
PULPROG	zgpg30
TD	65536
SOLVENT	CDCl3
NS	5120
DS	4
SWH	30120.482
FIDRES	0.919204
AQ	1.0878976
RG	101
DW	16.600
DE	6.50
TE	298.0
D1	2.00000000
D11	0.03000000
TD0	1
SFO1	125.7703643
NUC1	13C
P0	3.33
P1	10.00
PLW1	81.01100000
SFO2	500.1320005
NUC2	1H
CPDPRG2	waltz65
PCPD2	80.00
PLW2	22.34400000
PLW12	0.34912000
PLW13	0.17561000

F2 - Processing parameters

SI	32768
SF	125.7577885
WDW	EM
SSB	0
LB	1.00
GB	0
PC	1.40

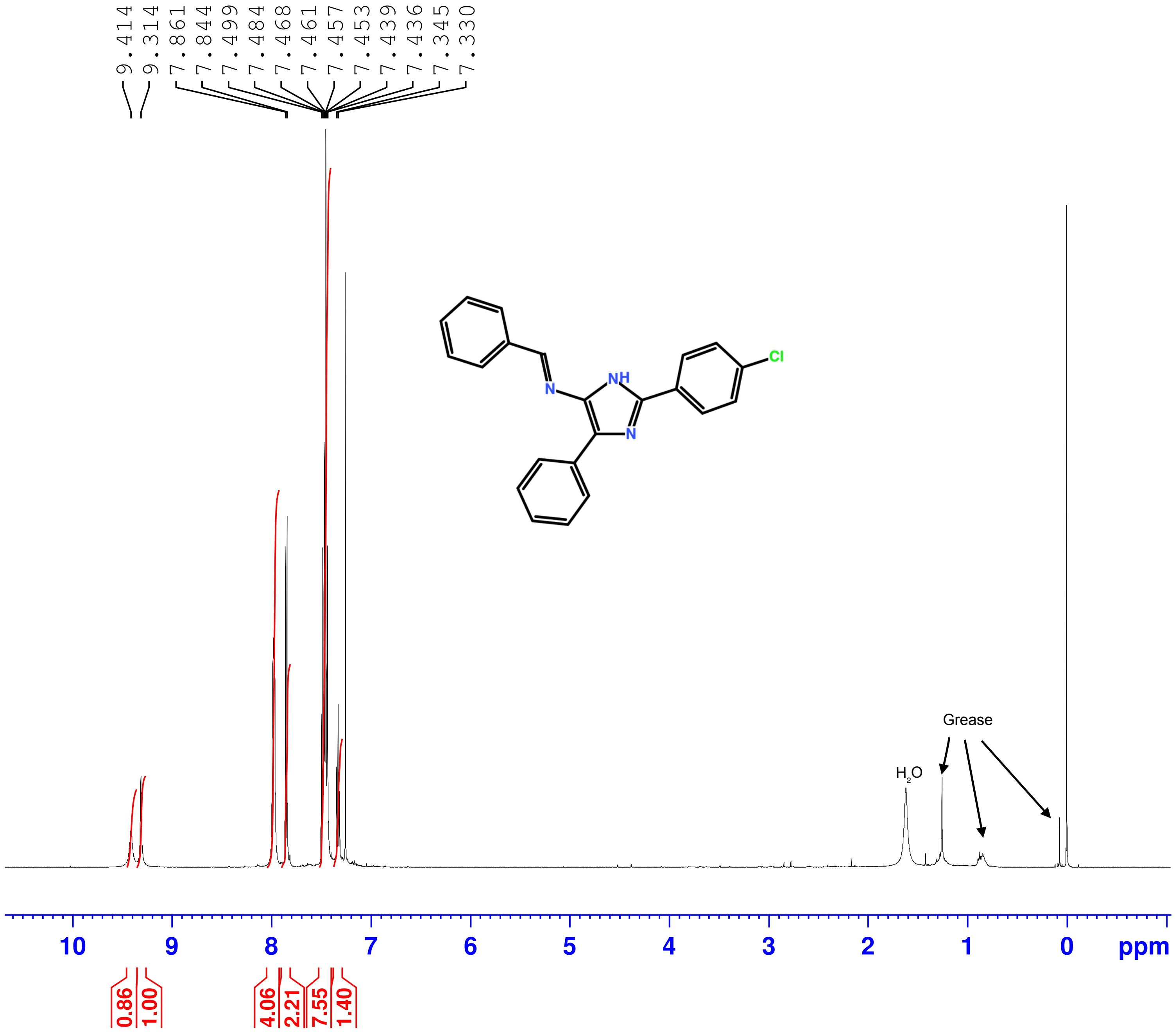

Current Data Parameters
NAME GBB-chloro-dry
EXPNO 1
PROCNO 1

F2 - Acquisition Parameters
Date_ 20250505
Time 22.43
INSTRUM Avance
PROBHD Z173362_0026 (PI HR-
PULPROG zg30
TD 65536
SOLVENT CDCl3
NS 16
DS 2
SWH 10000.000
FIDRES 0.305176
AQ 3.2768000
RG 101
DW 50.000
DE 10.79
TE 298.0
D1 1.00000000
TD0 1
SFO1 500.1330883
NUC1 1H
P0 3.33
P1 10.00
PLW1 22.34400000

F2 - Processing parameters
SI 65536
SF 500.1300140
WDW EM
SSB 0
LB 0.30
GB 0
PC 1.00

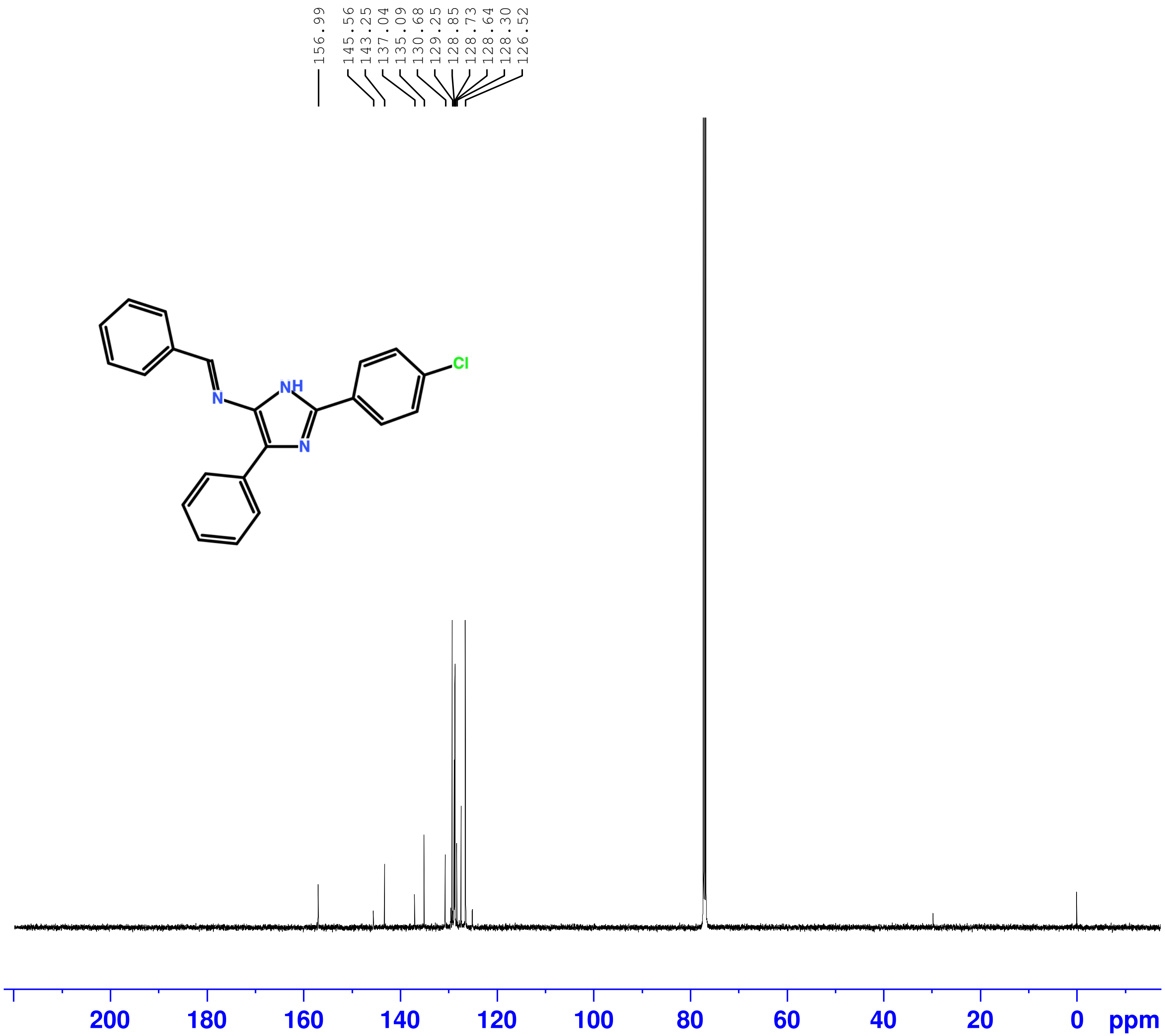

Current Data Parameters

NAME	GBB-chloro-dry
EXPNO	2
PROCNO	1

F2 - Acquisition Parameters

Date_	20250506
Time	3.13
INSTRUM	Avance
PROBHD	Z173362_0026 (PI HR-
PULPROG	zgpg30
TD	65536
SOLVENT	CDCl3
NS	5120
DS	4
SWH	30120.482
FIDRES	0.919204
AQ	1.0878976
RG	101
DW	16.600
DE	6.50
TE	298.0
D1	2.00000000
D11	0.03000000
TD0	1
SFO1	125.7703643
NUC1	13C
P0	3.33
P1	10.00
PLW1	81.01100000
SFO2	500.1320005
NUC2	1H
CPDPRG2	waltz65
PCPD2	80.00
PLW2	22.34400000
PLW12	0.34912000
PLW13	0.17561000

F2 - Processing parameters

SI	32768
SF	125.7577902
WDW	EM
SSB	0
LB	1.00
GB	0
PC	1.40

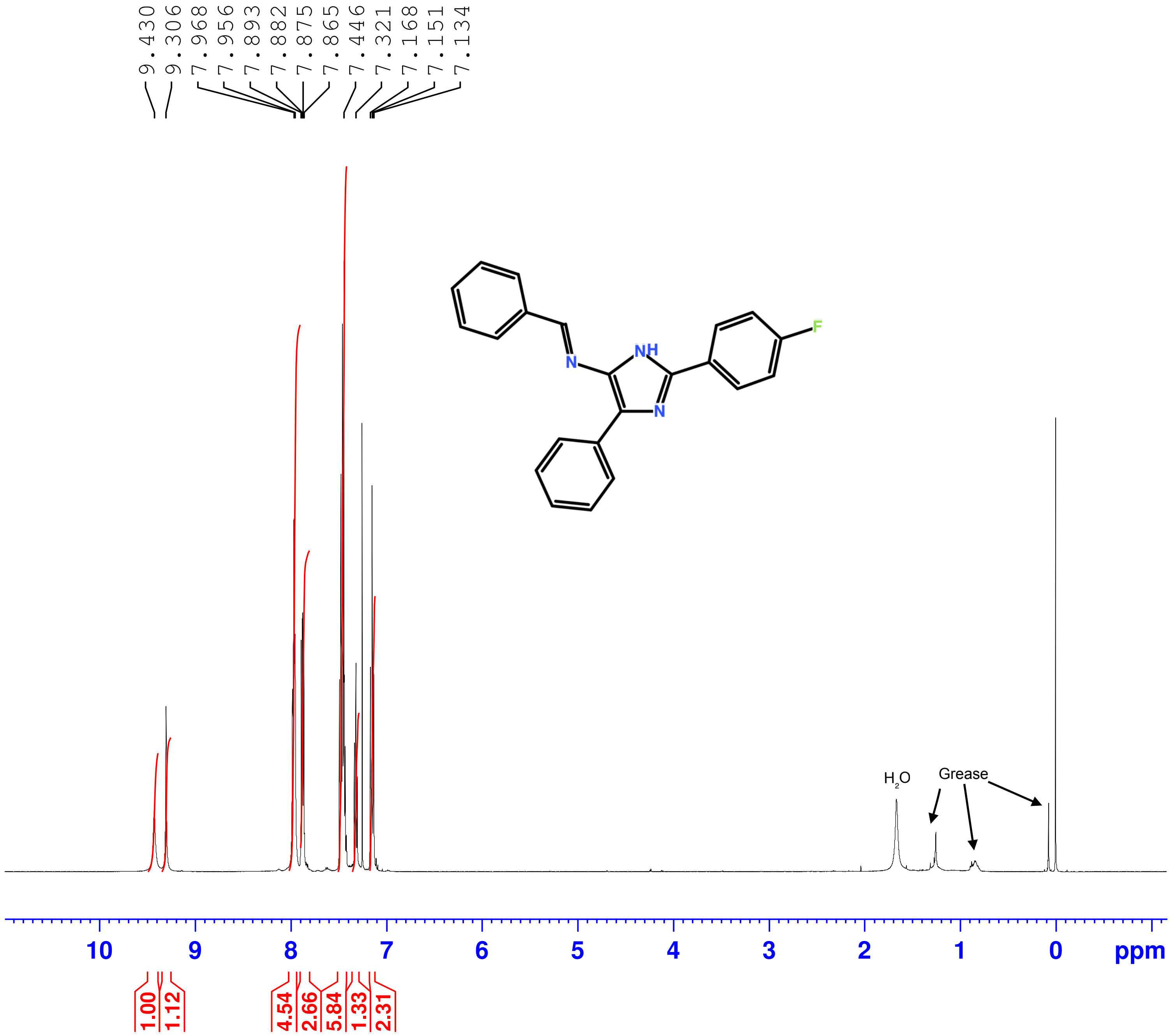

BRUKER

```
Current Data Parameters
NAME                GBB-fluoro-dry
EXPNO                            1
PROCNO                           1

F2 - Acquisition Parameters
Date_                     20250505
Time                         17.16
INSTRUM                     Avance
PROBHD      Z173362_0026 (PI HR-
PULPROG                       zg30
TD                           65536
SOLVENT                      CDCl3
NS                              16
DS                               2
SWH                      10000.000
FIDRES                    0.305176
AQ                       3.2768000
RG                             101
DW                          50.000
DE                           10.79
TE                           298.0
D1                      1.00000000
TD0                              1
SFO1                   500.1330883
NUC1                            1H
P0                            3.33
P1                           10.00
PLW1                   22.34400000

F2 - Processing parameters
SI                           65536
SF                     500.1300157
WDW                             EM
SSB                              0
LB                            0.30
GB                               0
PC                            1.00
```

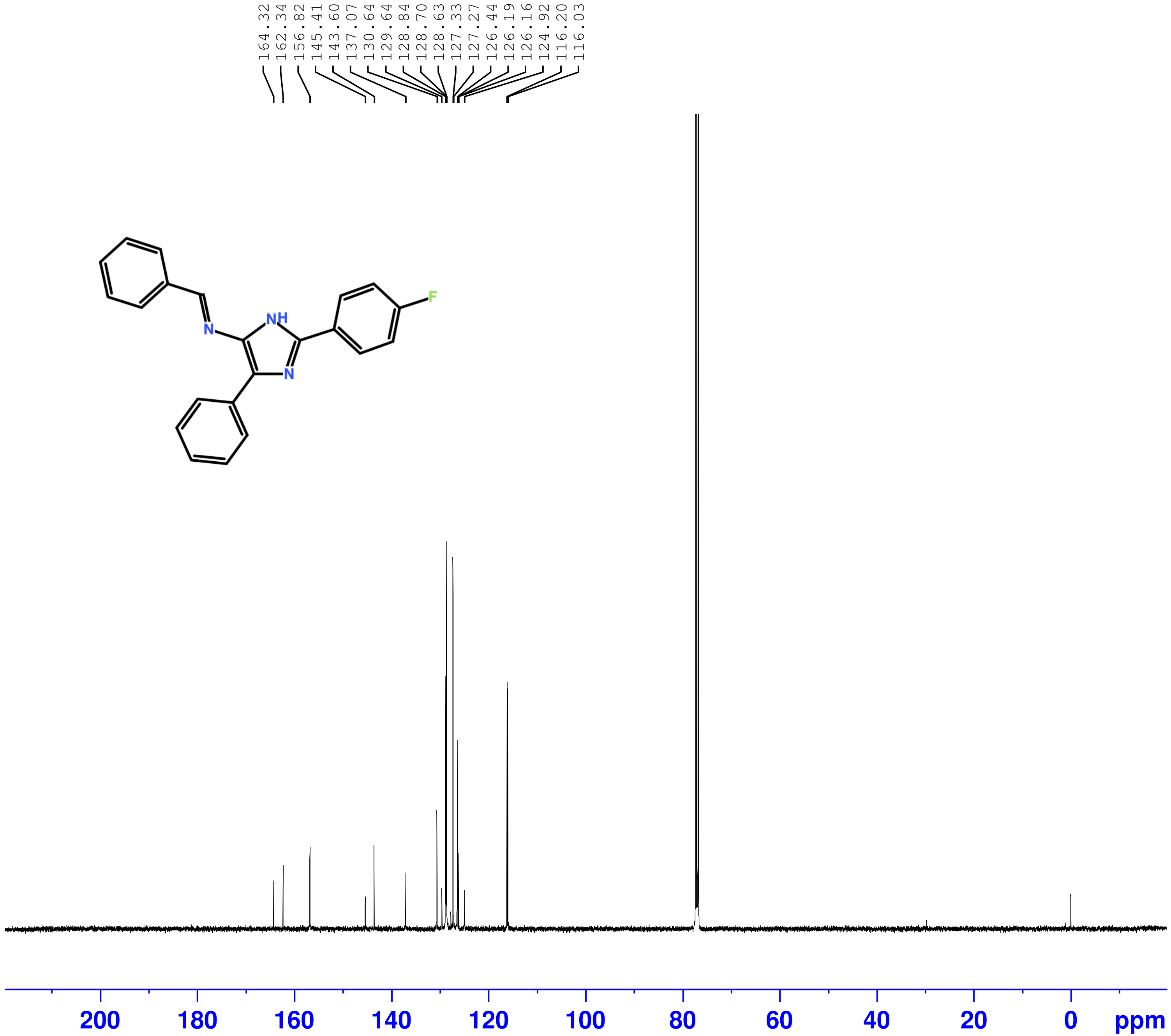

Current Data Parameters

NAME	GBB-fluoro-dry
EXPNO	2
PROCNO	1

F2 - Acquisition Parameters

Date_	20250505
Time	21.47
INSTRUM	Avance
PROBHD	Z173362_0026 (PI HR-
PULPROG	zgpg30
TD	65536
SOLVENT	CDCl3
NS	5120
DS	4
SWH	30120.482
FIDRES	0.919204
AQ	1.0878976
RG	101
DW	16.600
DE	6.50
TE	298.0
D1	2.00000000
D11	0.03000000
TD0	1
SFO1	125.7703643
NUC1	13C
P0	3.33
P1	10.00
PLW1	81.01100000
SFO2	500.1320005
NUC2	1H
CPDPRG2	waltz65
PCPD2	80.00
PLW2	22.34400000
PLW12	0.34912000
PLW13	0.17561000

F2 - Processing parameters

SI	32768
SF	125.7577906
WDW	EM
SSB	0
LB	1.00
GB	0
PC	1.40

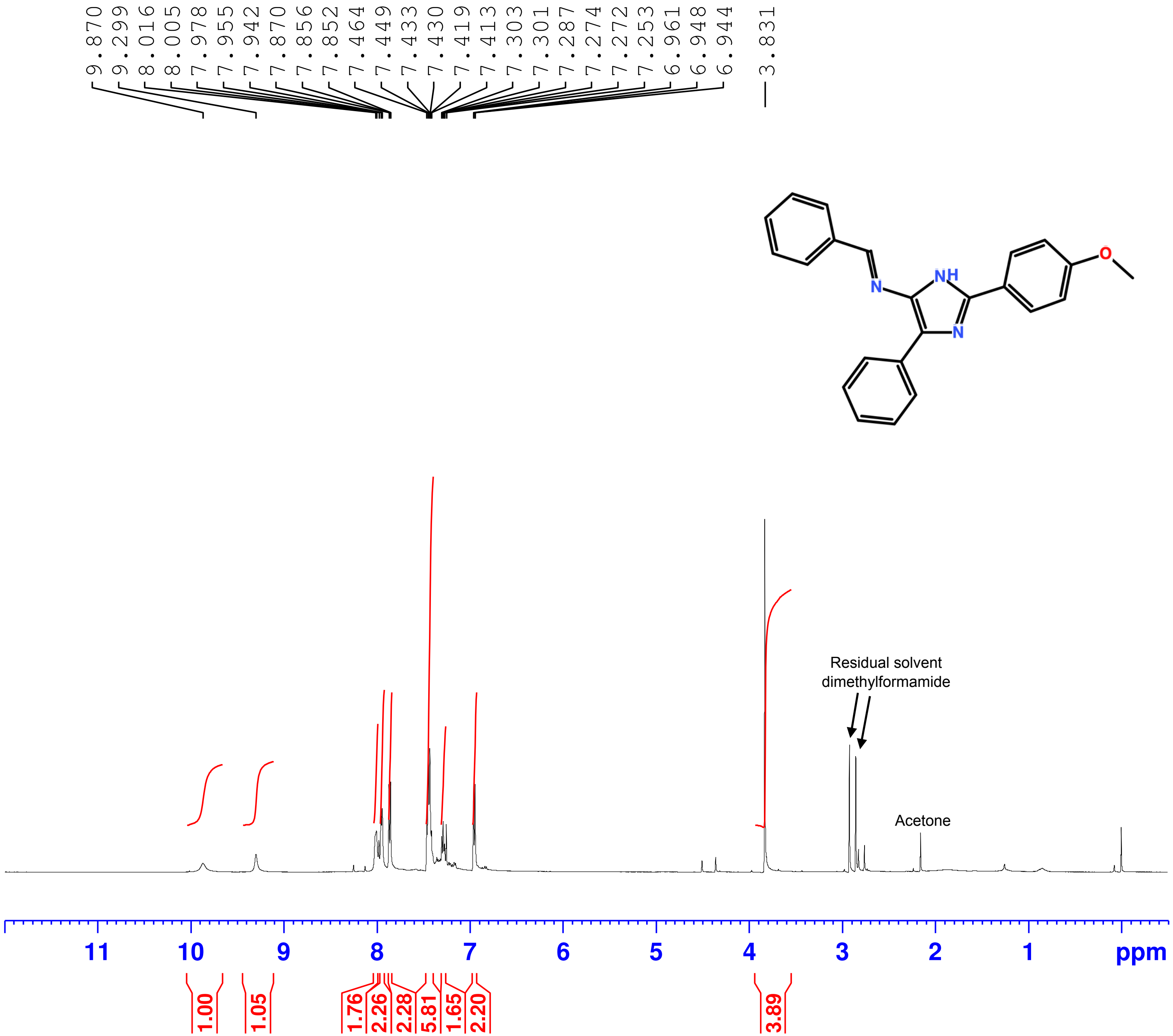

Current Data Parameters
NAME methoxy bami 2
EXPNO 2
PROCNO 1

F2 - Acquisition Parameters

Date_	20250522
Time	8.46
INSTRUM	Avance
PROBHD	Z173362_0026 (PI HR-
PULPROG	zg30
TD	65536
SOLVENT	CDCl3
NS	16
DS	2
SWH	10000.000
FIDRES	0.305176
AQ	3.2768000
RG	101
DW	50.000
DE	10.79
TE	298.0
D1	1.00000000
TD0	1
SFO1	500.1330883
NUC1	1H
P0	3.33
P1	10.00
PLW1	22.34400000

F2 - Processing parameters

SI	65536
SF	500.1300150
WDW	EM
SSB	0
LB	0.30
GB	0
PC	1.00

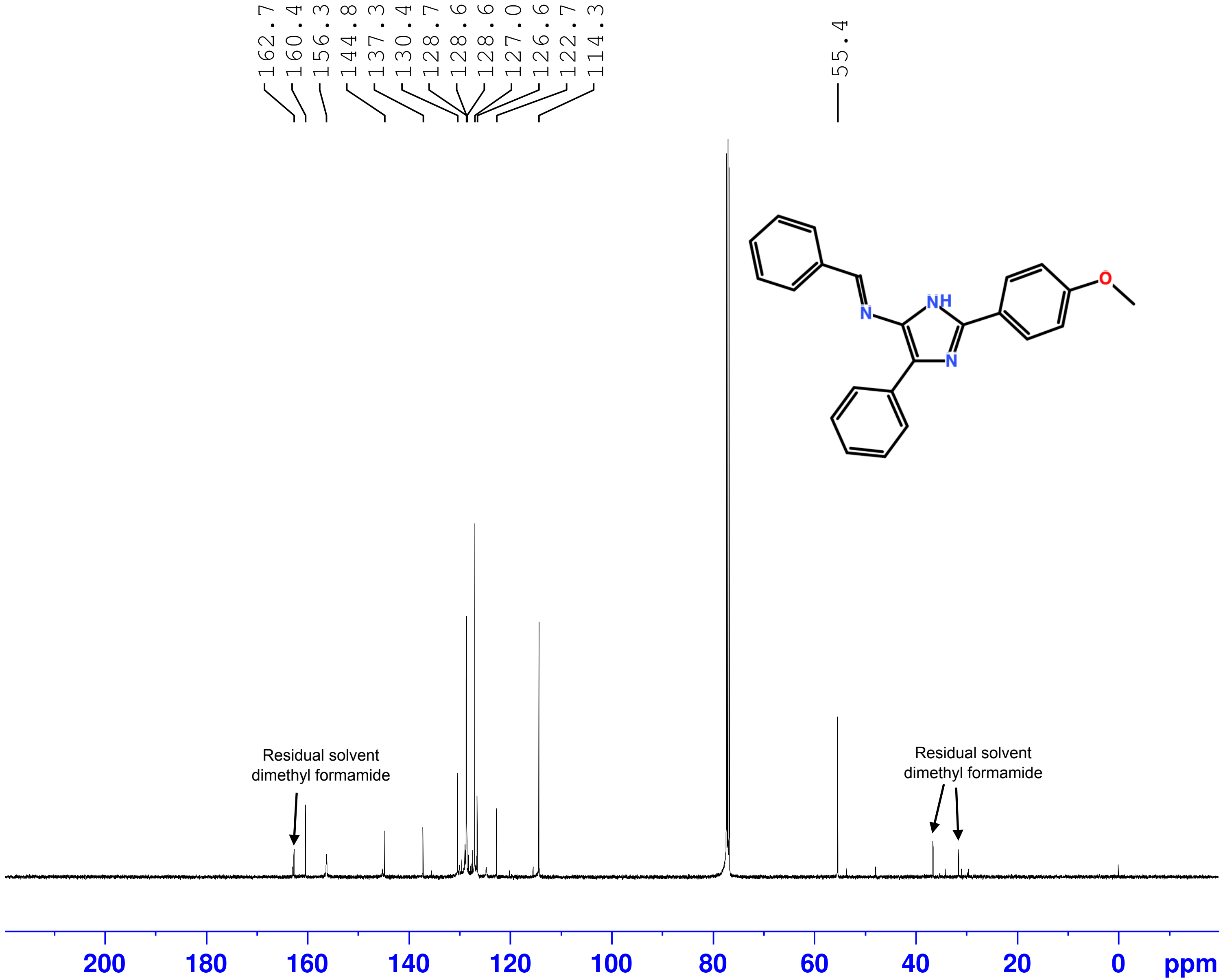

Current Data Parameters

NAME	methoxy bami 2
EXPNO	1
PROCNO	1

F2 - Acquisition Parameters

Date_	20250521
Time	19.58
INSTRUM	Avance
PROBHD	Z173362_0026 (PI HR-
PULPROG	zgpg30
TD	65536
SOLVENT	CDCl3
NS	5000
DS	4
SWH	30120.482
FIDRES	0.919204
AQ	1.0878976
RG	101
DW	16.600
DE	6.50
TE	298.0
D1	2.00000000
D11	0.03000000
TD0	1
SFO1	125.7703643
NUC1	13C
P0	3.33
P1	10.00
PLW1	81.01100000
SFO2	500.1320005
NUC2	1H
CPDPRG2	waltz65
PCPD2	80.00
PLW2	22.34400000
PLW12	0.34912000
PLW13	0.17561000

F2 - Processing parameters

SI	32768
SF	125.7577885
WDW	EM
SSB	0
LB	1.00
GB	0
PC	1.40

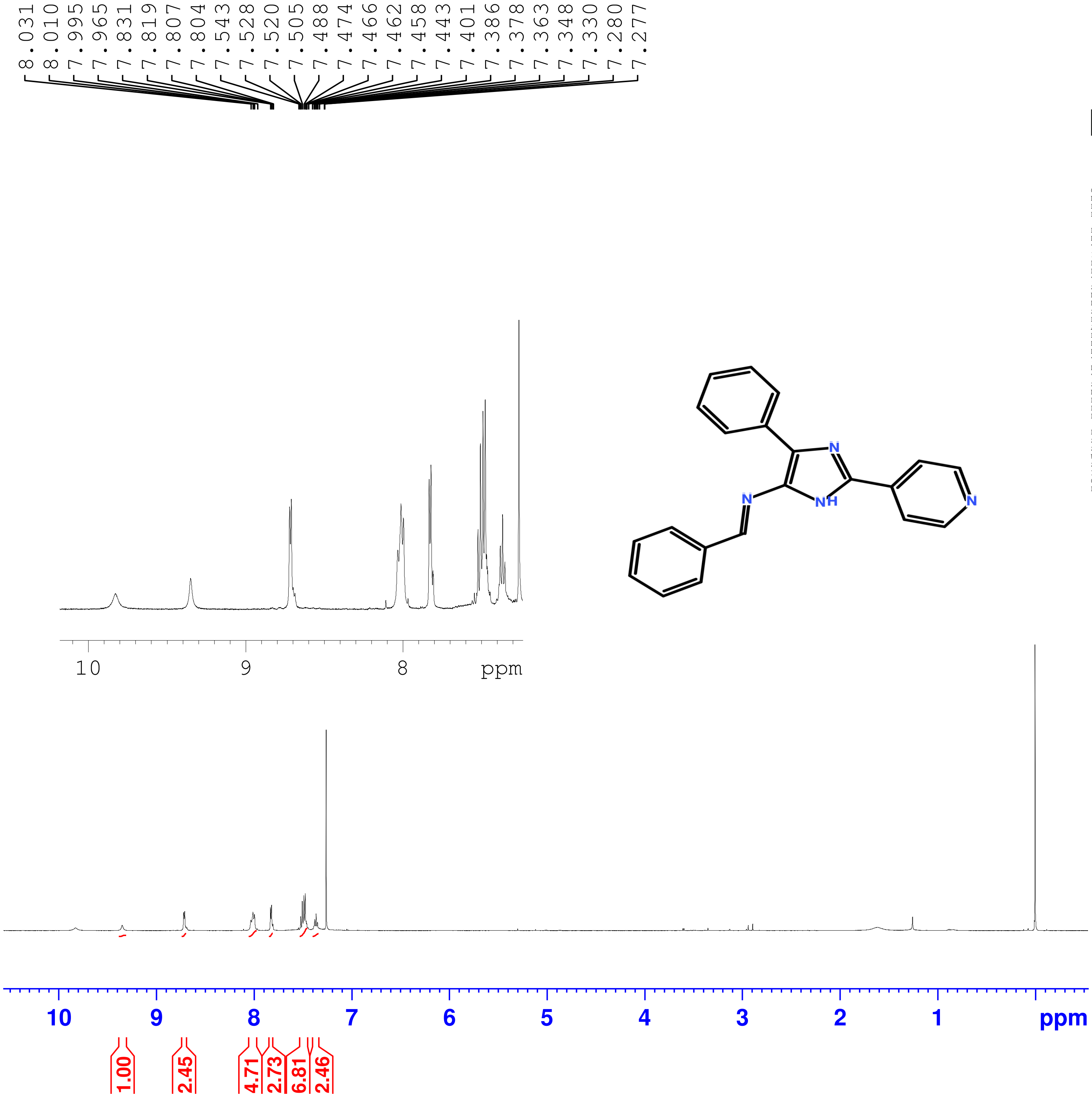


```
Current Data Parameters
NAME        HT-GBB-PIMI-6-2-26-GroupB
EXPNO                   1
PROCNO                  1

F2 - Acquisition Parameters
Date_            20260605
Time                10.16
INSTRUM            Avance
PROBHD      Z173362_0026 (PI HR-TBO500S1-BBF/H/F/D-5.0-Z FB)
PULPROG              zg30
TD                  65536
SOLVENT             CDCl3
NS                     16
DS                      2
SWH             10000.000 Hz
FIDRES           0.305176 Hz
AQ              3.2768000 sec
RG                    101
DW                 50.000 usec
DE                  10.79 usec
TE                  298.0 K
D1             1.00000000 sec
TD0                     1
SFO1          500.1330883 MHz
NUC1                   1H
P0                   3.33 usec
P1                  10.00 usec
PLW1          22.34400000 W

F2 - Processing parameters
SI                  65536
SF            500.1300130 MHz
WDW                    EM
SSB                     0
LB                   0.30 Hz
GB                      0
PC                   1.00
```

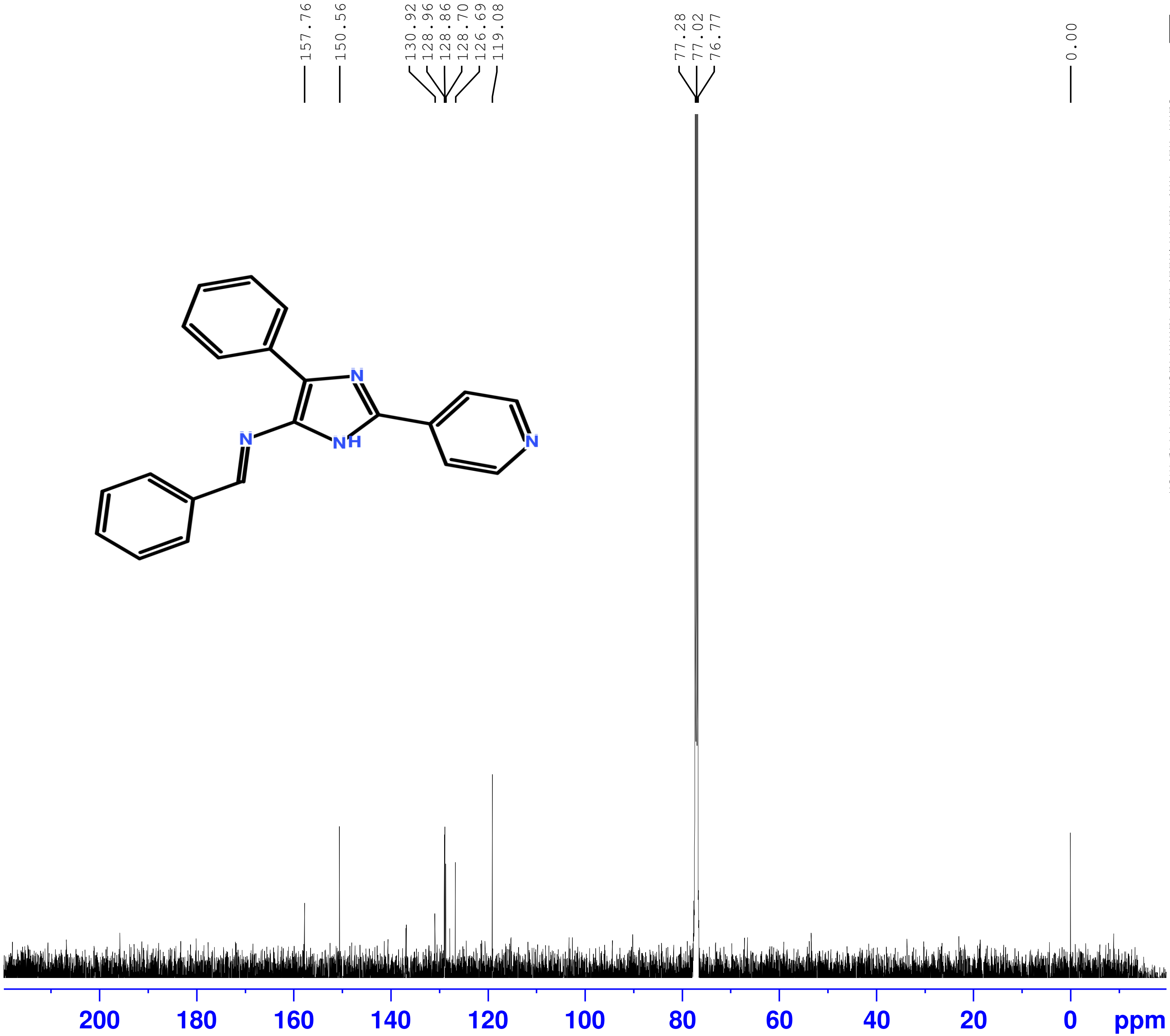


```
Current Data Parameters
NAME              KJW-GBB-4-17-25
EXPNO                           3
PROCNO                          1

F2 - Acquisition Parameters
Date_                    20250421
Time                        20.21
INSTRUM                    Avance
PROBHD     Z173362_0026 (PI HR-TBO500S1-BBF/H/F/D-5.0-Z FB)
PULPROG                   zgpg30
TD                          65536
SOLVENT                     CDCl3
NS                           3072
DS                              4
SWH                     30120.482 Hz
FIDRES                   0.919204 Hz
AQ                      1.0878976 sec
RG                            101
DW                         16.600 usec
DE                           6.50 usec
TE                          298.0 K
D1                     2.00000000 sec
D11                    0.03000000 sec
TD0                             1
SFO1                  125.7703643 MHz
NUC1                          13C
P0                           3.33 usec
P1                          10.00 usec
PLW1                  81.01100000 W
SFO2                  500.1320005 MHz
NUC2                           1H
CPDPRG2                   waltz65
PCPD2                       80.00 usec
PLW2                  22.34400000 W
PLW12                  0.34912000 W
PLW13                  0.17561000 W

F2 - Processing parameters
SI                          32768
SF                    125.7577885 MHz
WDW                            EM
SSB                             0
LB                           1.00 Hz
GB                              0
PC                           1.40
```

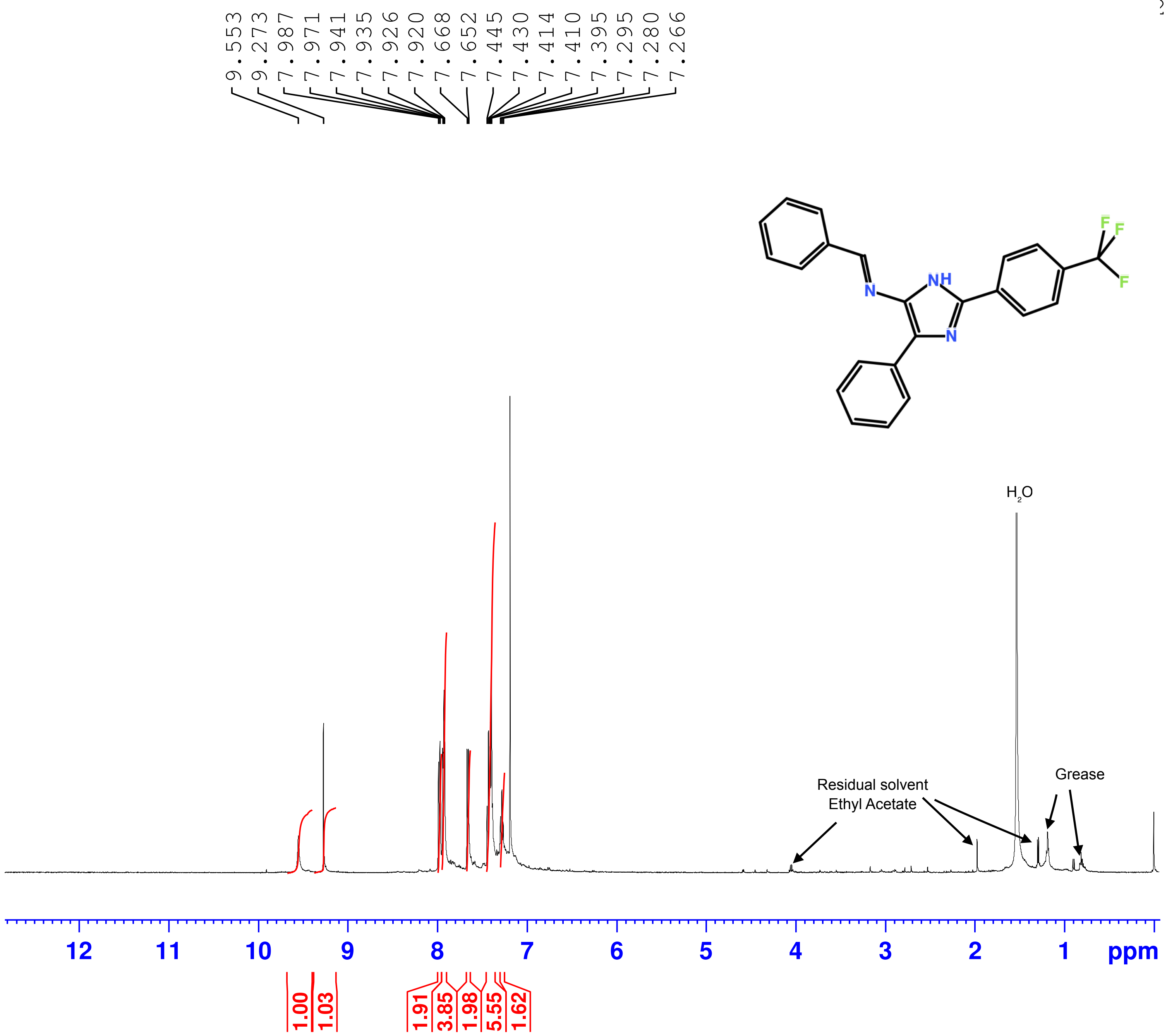

Current Data Parameters

NAME	GBB-6-10-25-C
EXPNO	1
PROCNO	1

F2 - Acquisition Parameters

Date_	20250618
Time	15.38
INSTRUM	Avance
PROBHD	Z173362_0026 (PI HR-
PULPROG	zg30
TD	65536
SOLVENT	CDCl3
NS	16
DS	2
SWH	10000.000
FIDRES	0.305176
AQ	3.2768000
RG	101
DW	50.000
DE	10.79
TE	298.0
D1	1.00000000
TD0	1
SFO1	500.1330883
NUC1	1H
P0	3.33
P1	10.00
PLW1	22.34400000

F2 - Processing parameters

SI	65536
SF	500.1300474
WDW	EM
SSB	0
LB	0.30
GB	0
PC	1.00

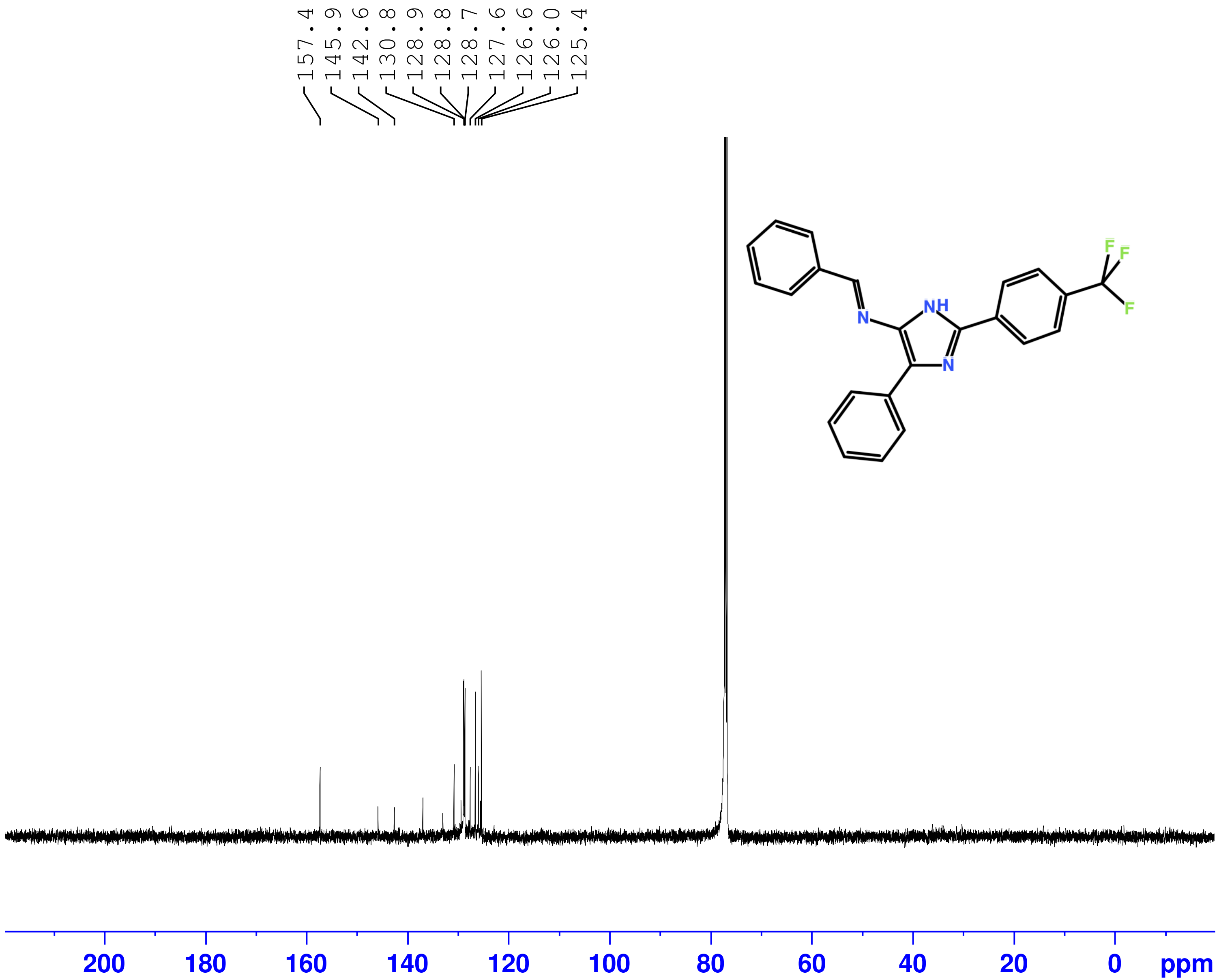

Current Data Parameters

NAME	GBB-6-10-25-C
EXPNO	3
PROCNO	1

F2 - Acquisition Parameters

Date_	20250618
Time	23.34
INSTRUM	Avance
PROBHD	Z173362_0026 (PI HR-
PULPROG	zgpg30
TD	65536
SOLVENT	CDCl3
NS	5120
DS	4
SWH	30120.482
FIDRES	0.919204
AQ	1.0878976
RG	101
DW	16.600
DE	6.50
TE	298.0
D1	2.00000000
D11	0.03000000
TD0	1
SFO1	125.7703643
NUC1	13C
P0	3.33
P1	10.00
PLW1	81.01100000
SFO2	500.1320005
NUC2	1H
CPDPRG2	waltz65
PCPD2	80.00
PLW2	22.34400000
PLW12	0.34912000
PLW13	0.17561000

F2 - Processing parameters

SI	32768
SF	125.7577885
WDW	EM
SSB	0
LB	1.00
GB	0
PC	1.40

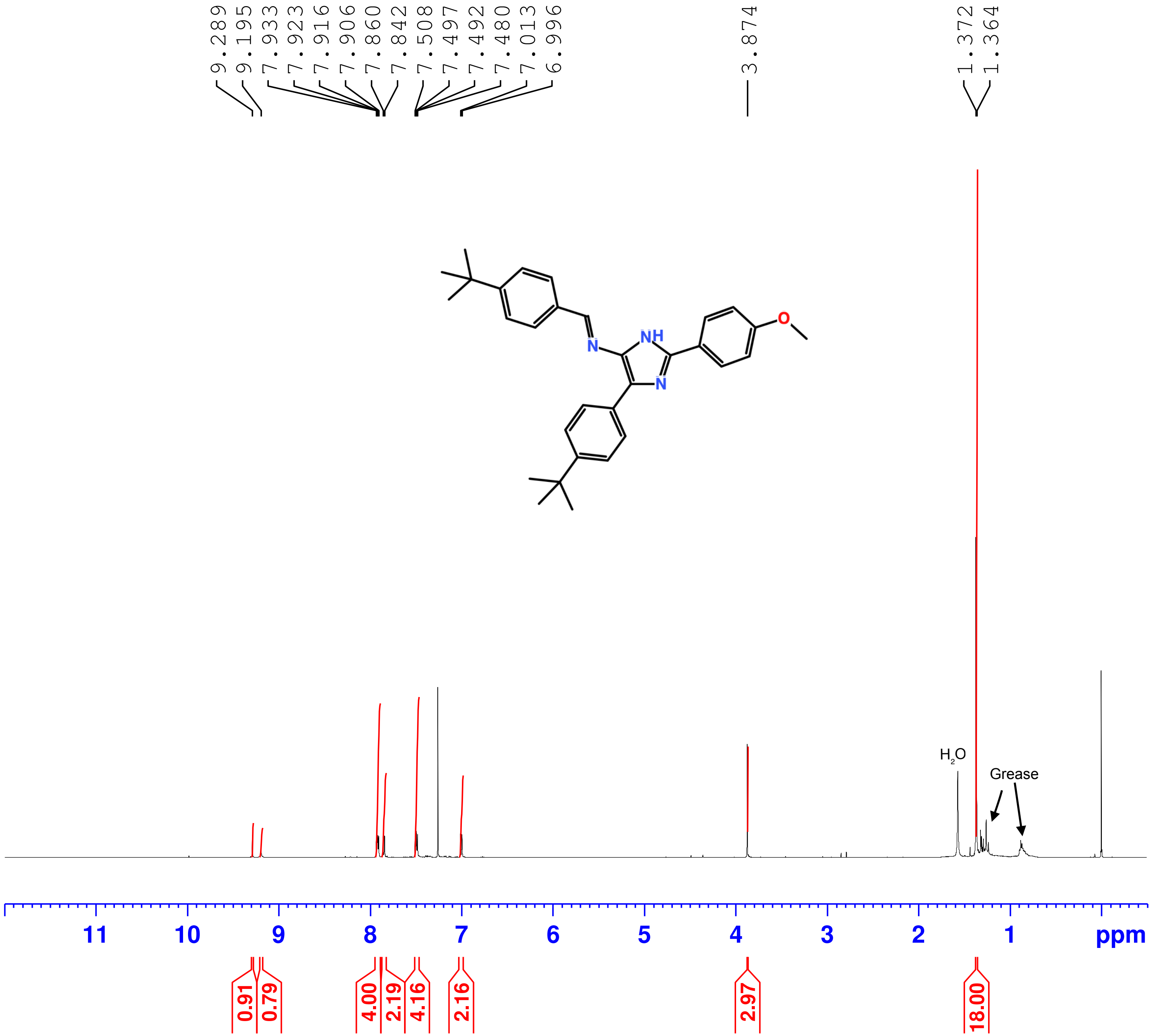


```
Current Data Parameters
NAME          t-bad & methoxy bam
EXPNO                           1
PROCNO                          1

F2 - Acquisition Parameters
Date_                    20250521
Time                        10.39
INSTRUM                    Avance
PROBHD      Z173362_0026 (PI HR-
PULPROG                      zg30
TD                          65536
SOLVENT                     CDCl3
NS                             16
DS                              2
SWH                     10000.000
FIDRES                   0.305176
AQ                      3.2768000
RG                            101
DW                         50.000
DE                          10.79
TE                          298.0
D1                     1.00000000
TD0                             1
SFO1                  500.1330883
NUC1                           1H
P0                           3.33
P1                          10.00
PLW1                  22.34400000

F2 - Processing parameters
SI                          65536
SF                    500.1300123
WDW                            EM
SSB                             0
LB                           0.30
GB                              0
PC                           1.00
```

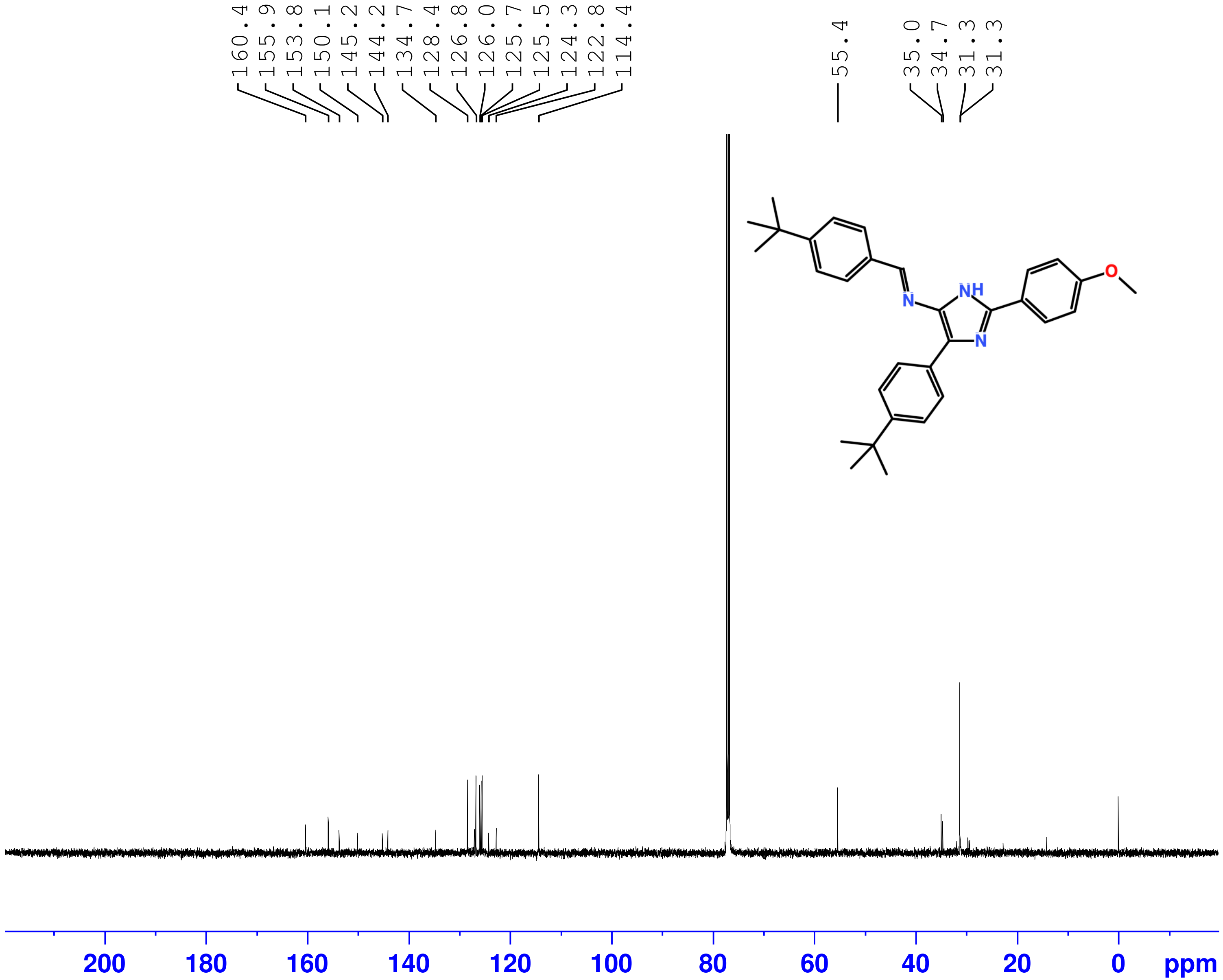

Current Data Parameters
NAME t-bad & methoxy bam
EXPNO 2
PROCNO 1

F2 - Acquisition Parameters
Date_ 20250521
Time 15.28
INSTRUM Avance
PROBHD Z173362_0026 (PI HR-
PULPROG zgpg30
TD 65536
SOLVENT CDCl3
NS 5120
DS 4
SWH 30120.482
FIDRES 0.919204
AQ 1.0878976
RG 101
DW 16.600
DE 6.50
TE 298.0
D1 2.00000000
D11 0.03000000
TD0 1
SFO1 125.7703643
NUC1 13C
P0 3.33
P1 10.00
PLW1 81.01100000
SFO2 500.1320005
NUC2 1H
CPDPRG2 waltz65
PCPD2 80.00
PLW2 22.34400000
PLW12 0.34912000
PLW13 0.17561000

F2 - Processing parameters
SI 32768
SF 125.7577895
WDW EM
SSB 0
LB 1.00
GB 0
PC 1.40

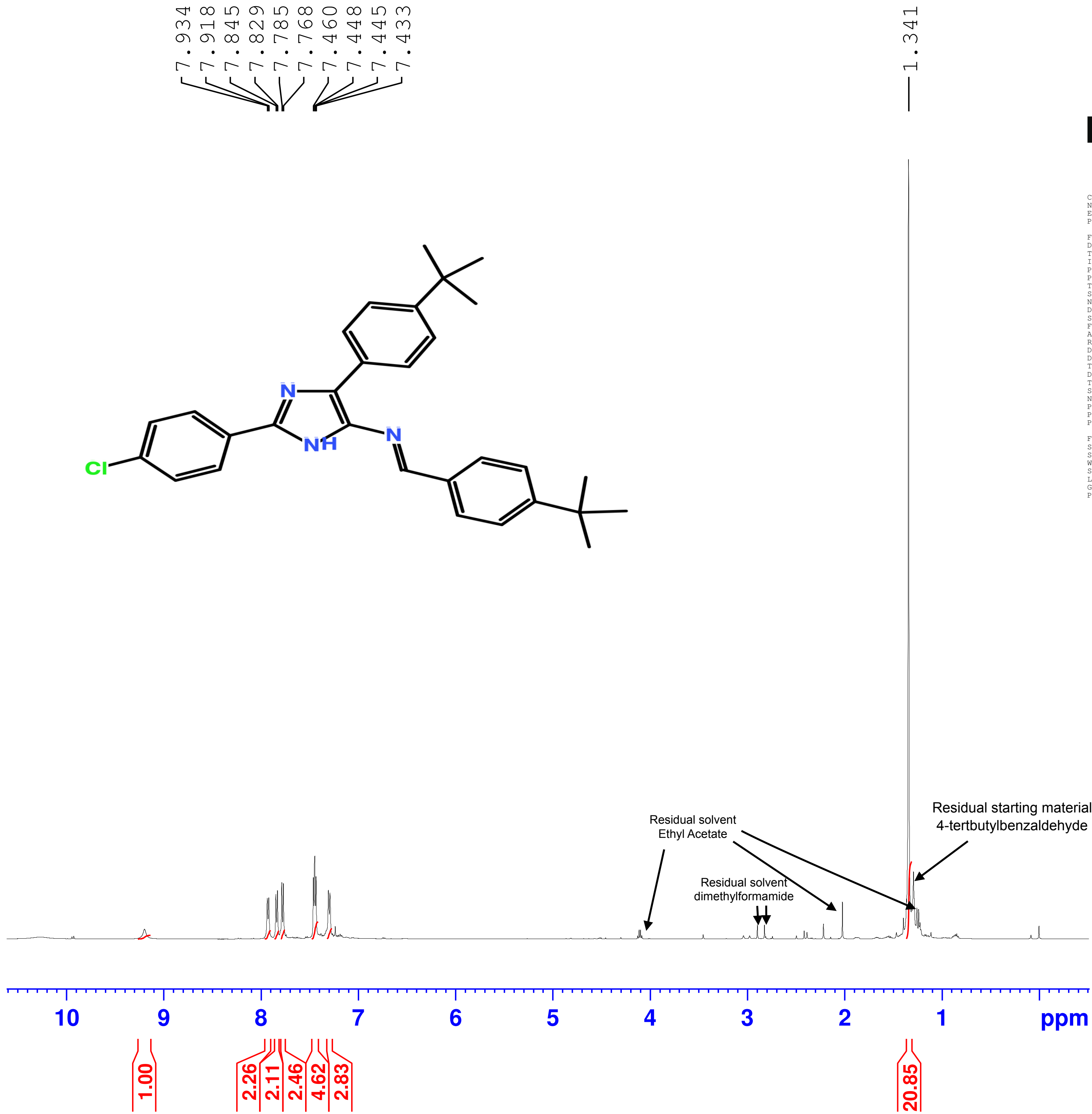


```
Current Data Parameters
NAME          GBB-Cl-BAMI + tBu
EXPNO                     1
PROCNO                    1

F2 - Acquisition Parameters
Date_              20260305
Time                  20.00
INSTRUM              Avance
PROBHD     Z173362_0026 (PI HR-TBO500S1-BBF/H/F/D-5.0-Z FB)
PULPROG                zg30
TD                    65536
SOLVENT               CDCl3
NS                       16
DS                        2
SWH               10000.000 Hz
FIDRES             0.305176 Hz
AQ                3.2768000 sec
RG                     45.2
DW                   50.000 usec
DE                    10.79 usec
TE                    298.0 K
D1               1.00000000 sec
TD0                       1
SFO1            500.1330883 MHz
NUC1                     1H
P0                     3.33 usec
P1                    10.00 usec
PLW1            22.34400000 W

F2 - Processing parameters
SI                    65536
SF              500.1300240 MHz
WDW                      EM
SSB                       0
LB                     0.30 Hz
GB                        0
PC                     1.00
```

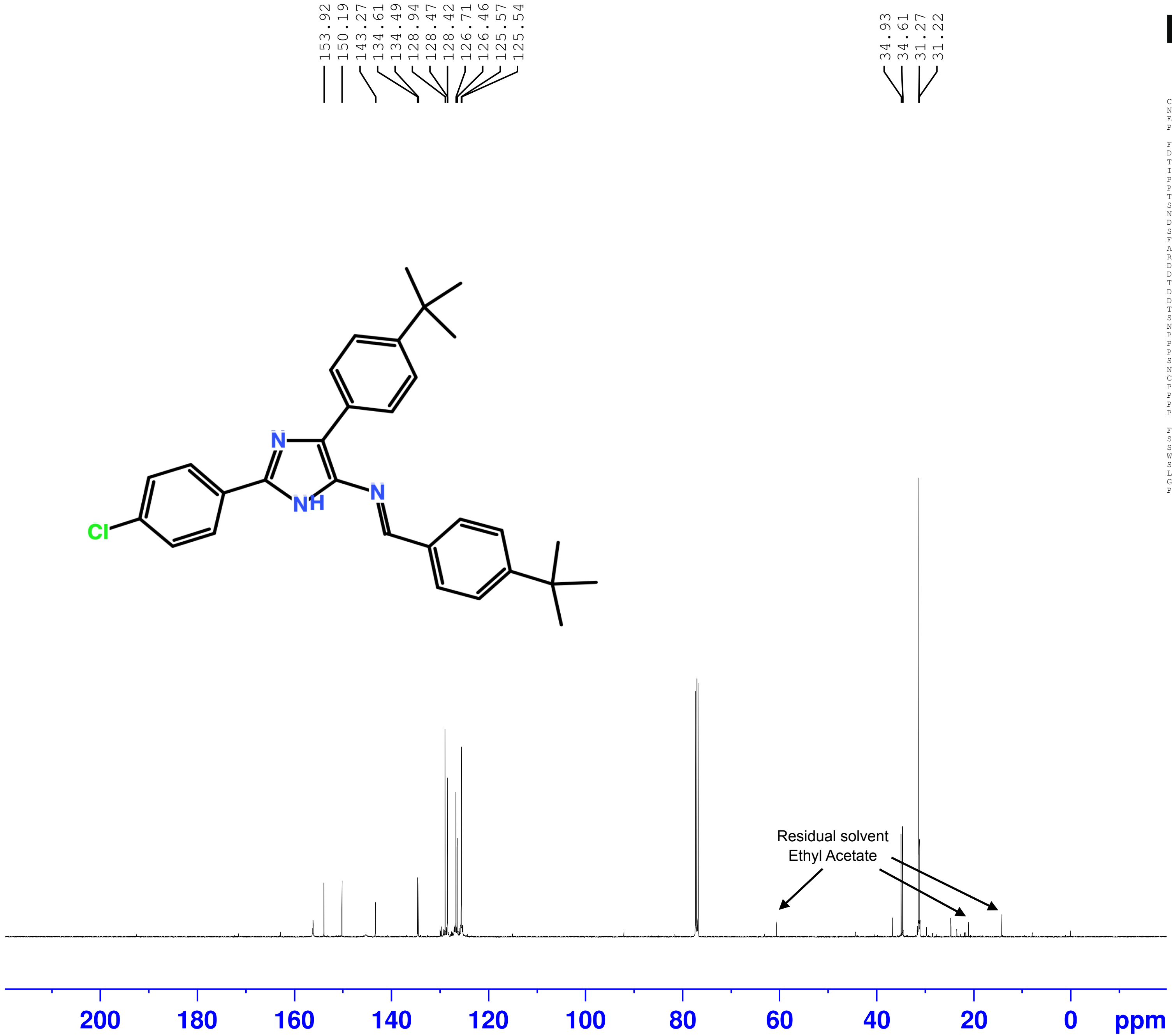


```
Current Data Parameters
NAME            GBB-Cl-BAMI + tBu
EXPNO                           2
PROCNO                          1

F2 - Acquisition Parameters
Date_                    20260306
Time                         0.32
INSTRUM                    Avance
PROBHD     Z173362_0026 (PI HR-TBO500S1-BBF/H/F/D-5.0-Z FB)
PULPROG                    zgpg30
TD                          65536
SOLVENT                     CDCl3
NS                           5120
DS                              4
SWH                     30120.482 Hz
FIDRES                   0.919204 Hz
AQ                      1.0878976 sec
RG                            101
DW                         16.600 usec
DE                           6.50 usec
TE                          298.0 K
D1                     2.00000000 sec
D11                    0.03000000 sec
TD0                             1
SFO1                  125.7703643 MHz
NUC1                          13C
P0                           3.33 usec
P1                          10.00 usec
PLW1                  81.01100000 W
SFO2                  500.1320005 MHz
NUC2                           1H
CPDPRG2                   waltz65
PCPD2                       80.00 usec
PLW2                  22.34400000 W
PLW12                  0.34912000 W
PLW13                  0.17561000 W

F2 - Processing parameters
SI                          32768
SF                    125.7577952 MHz
WDW                            EM
SSB                             0
LB                           1.00 Hz
GB                              0
PC                           1.40
```

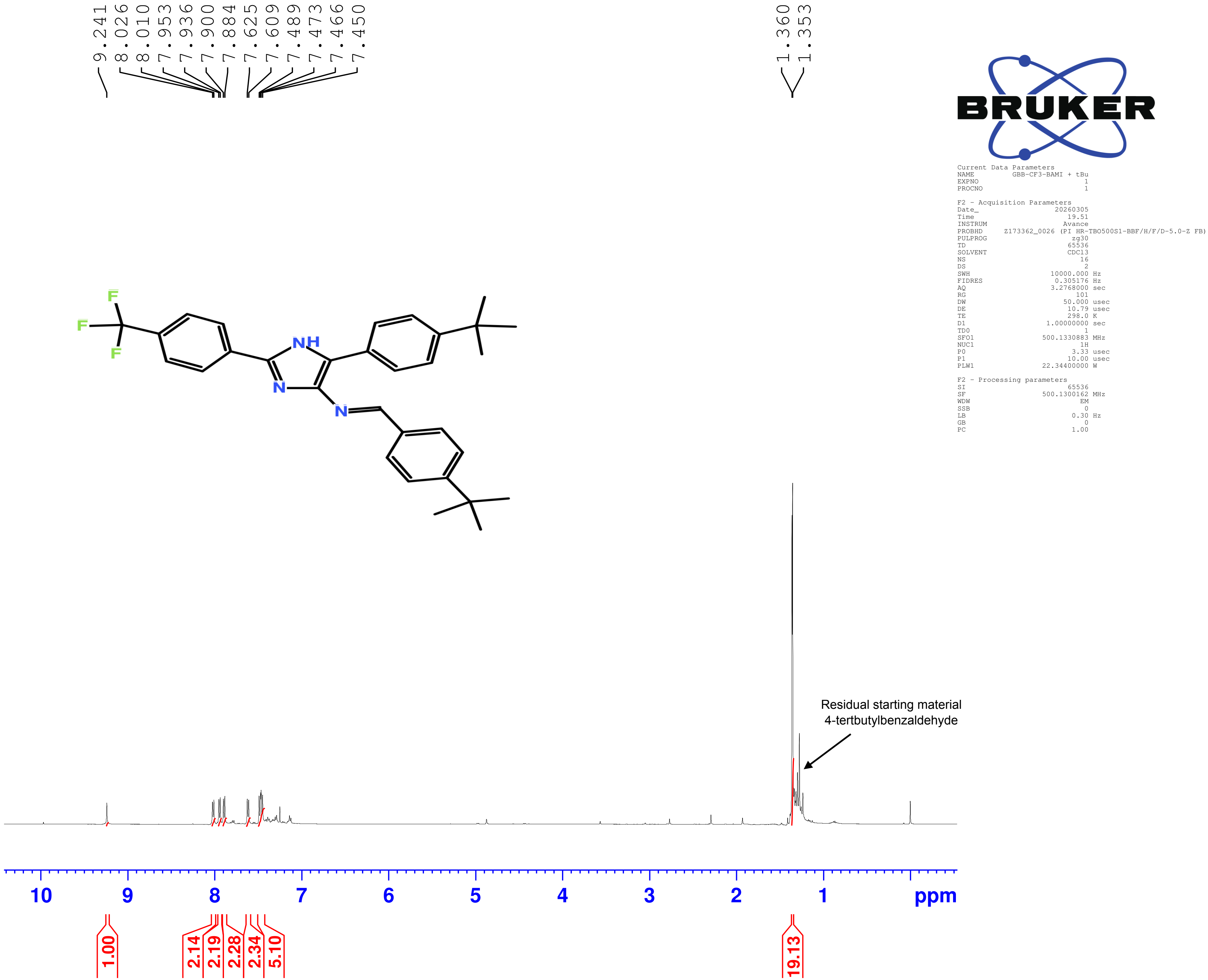
9.241
8.026
8.010
7.953
7.936
7.900
7.884
7.625
7.609
7.489
7.473
7.466
7.450
1.360
1.353
NH
N
N
F
F
F
Residual starting material
4-tertbutylbenzaldehyde
10
9
8
7
6
5
4
3
2
1
ppm
1.00
2.14
2.19
2.28
2.34
5.10
19.13
Current Data Parameters
NAME GBB-CF3-BAMI + tBu
EXPNO 1
PROCNO 1
F2 - Acquisition Parameters
Date_ 20260305
Time 19.51
INSTRUM Avance
PROBHD Z173362_0026 (PI HR-TBO500S1-BBF/H/F/D-5.0-Z FB)
PULPROG zg30
TD 65536
SOLVENT CDCl3
NS 16
DS 2
SWH 10000.000 Hz
FIDRES 0.305176 Hz
AQ 3.2768000 sec
RG 101
DW 50.000 usec
DE 10.79 usec
TE 298.0 K
D1 1.00000000 sec
TD0 1
SFO1 500.1330883 MHz
NUC1 1H
P0 3.33 usec
P1 10.00 usec
PLW1 22.34400000 W
F2 - Processing parameters
SI 65536
SF 500.1300162 MHz
WDW EM
SSB 0
LB 0.30 Hz
GB 0
PC 1.00

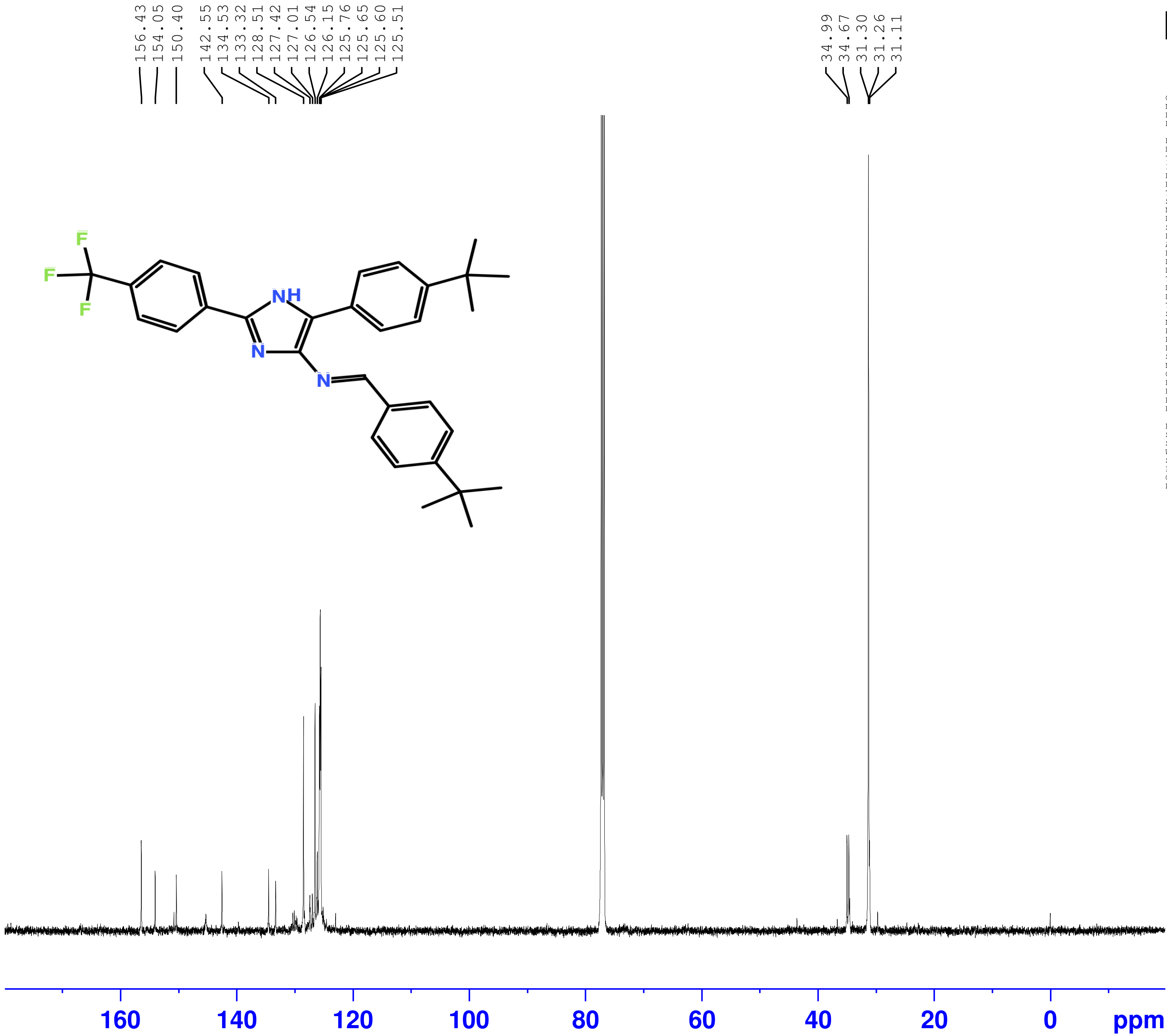


```
Current Data Parameters
NAME         GBB-CF3-BAMI + tBu
EXPNO                         3
PROCNO                        1

F2 - Acquisition Parameters
Date_                  20260306
Time                       5.05
INSTRUM                  Avance
PROBHD     Z173362_0026 (PI HR-TBO500S1-BBF/H/F/D-5.0-Z FB)
PULPROG                 zgpg30
TD                        65536
SOLVENT                   CDCl3
NS                         5120
DS                            4
SWH                   30120.482 Hz
FIDRES                 0.919204 Hz
AQ                    1.0878976 sec
RG                          101
DW                       16.600 usec
DE                         6.50 usec
TE                        298.0 K
D1                   2.00000000 sec
D11                  0.03000000 sec
TD0                           1
SFO1                125.7703643 MHz
NUC1                        13C
P0                         3.33 usec
P1                        10.00 usec
PLW1                81.01100000 W
SFO2                500.1320005 MHz
NUC2                         1H
CPDPRG2                 waltz65
PCPD2                     80.00 usec
PLW2                22.34400000 W
PLW12                0.34912000 W
PLW13                0.17561000 W

F2 - Processing parameters
SI                        32768
SF                  125.7577885 MHz
WDW                          EM
SSB                           0
LB                         1.00 Hz
GB                            0
PC                         1.40
```

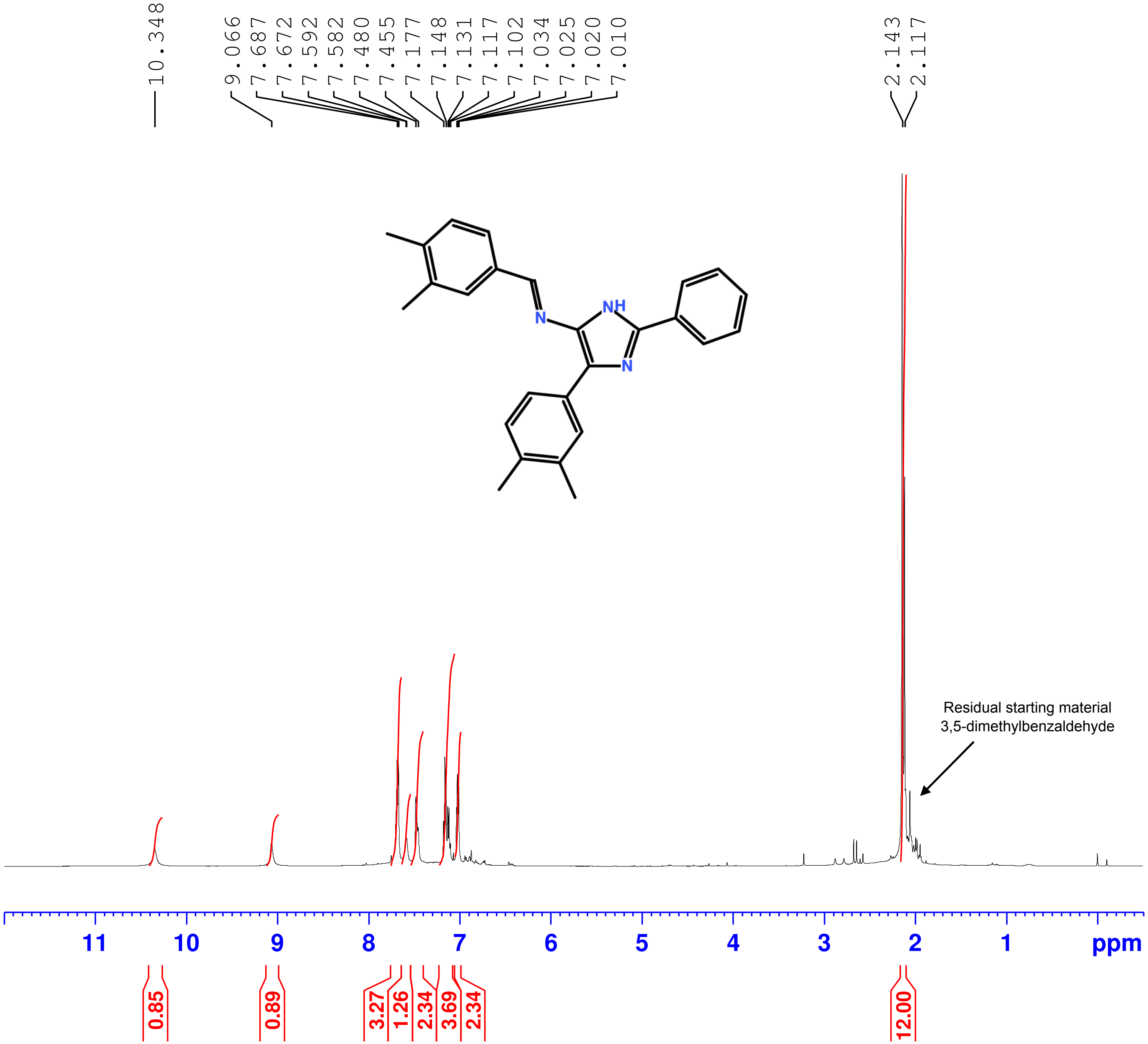


```
Current Data Parameters
NAME          GBB-3,4-dimethylbenz
EXPNO                            3
PROCNO                           1

F2 - Acquisition Parameters
Date_                     20250529
Time                         12.31
INSTRUM                     Avance
PROBHD      Z173362_0026 (PI HR-
PULPROG                       zg30
TD                           65536
SOLVENT                      CDCl3
NS                              16
DS                               2
SWH                      10000.000
FIDRES                    0.305176
AQ                       3.2768000
RG                              32
DW                          50.000
DE                           10.79
TE                           298.0
D1                      1.00000000
TD0                              1
SFO1                   500.1330883
NUC1                            1H
P0                            3.33
P1                           10.00
PLW1                   22.34400000

F2 - Processing parameters
SI                           65536
SF                     500.1301090
WDW                             EM
SSB                              0
LB                            0.30
GB                               0
PC                            1.00
```

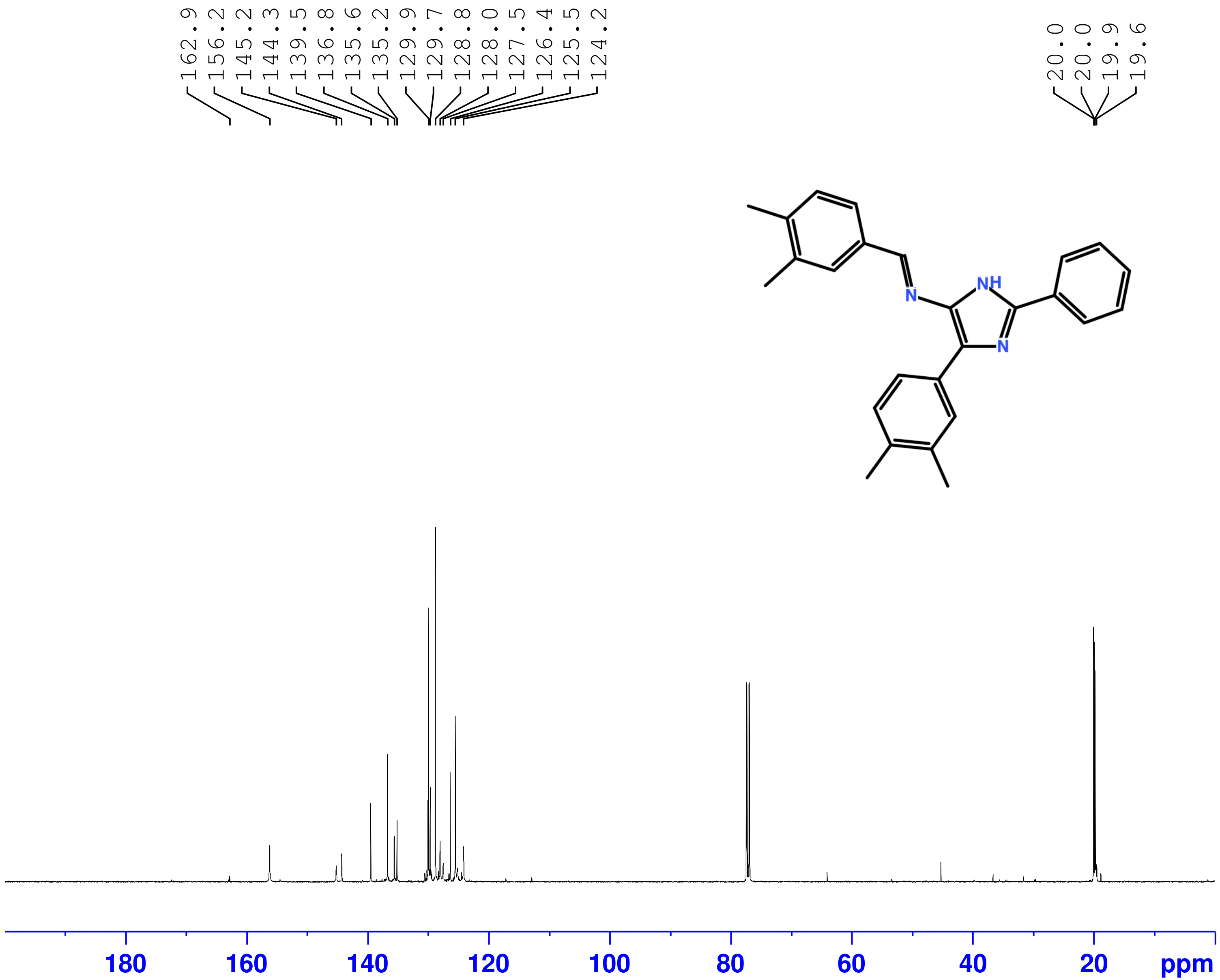

Current Data Parameters

NAME	GBB-3,4-dimethylbenz
EXPNO	5
PROCNO	1

F2 - Acquisition Parameters

Date_	20250529
Time	18.19
INSTRUM	Avance
PROBHD	Z173362_0026 (PI HR-
PULPROG	zgpg30
TD	65536
SOLVENT	CDCl3
NS	5120
DS	4
SWH	30120.482
FIDRES	0.919204
AQ	1.0878976
RG	101
DW	16.600
DE	6.50
TE	298.0
D1	2.00000000
D11	0.03000000
TD0	1
SFO1	125.7703643
NUC1	13C
P0	3.33
P1	10.00
PLW1	81.01100000
SFO2	500.1320005
NUC2	1H
CPDPRG2	waltz65
PCPD2	80.00
PLW2	22.34400000
PLW12	0.34912000
PLW13	0.17561000

F2 - Processing parameters

SI	32768
SF	125.7577885
WDW	EM
SSB	0
LB	1.00
GB	0
PC	1.40

===

Acq. Operator	: SYSTEM	Seq. Line :	1
Acq. Instrument	: LC	Location :	Vial 1
Injection Date	: 7/22/2026 11:54:13 AM	Inj :	5
		Inj Volume :	Manually

Sequence File : C:\CHEM32\1\SEQUENCE\KEM.S
Method : C:\CHEM32\1\METHODS\KAM.M
Last changed : 7/22/2026 11:48:47 AM by SYSTEM
(modified after loading)

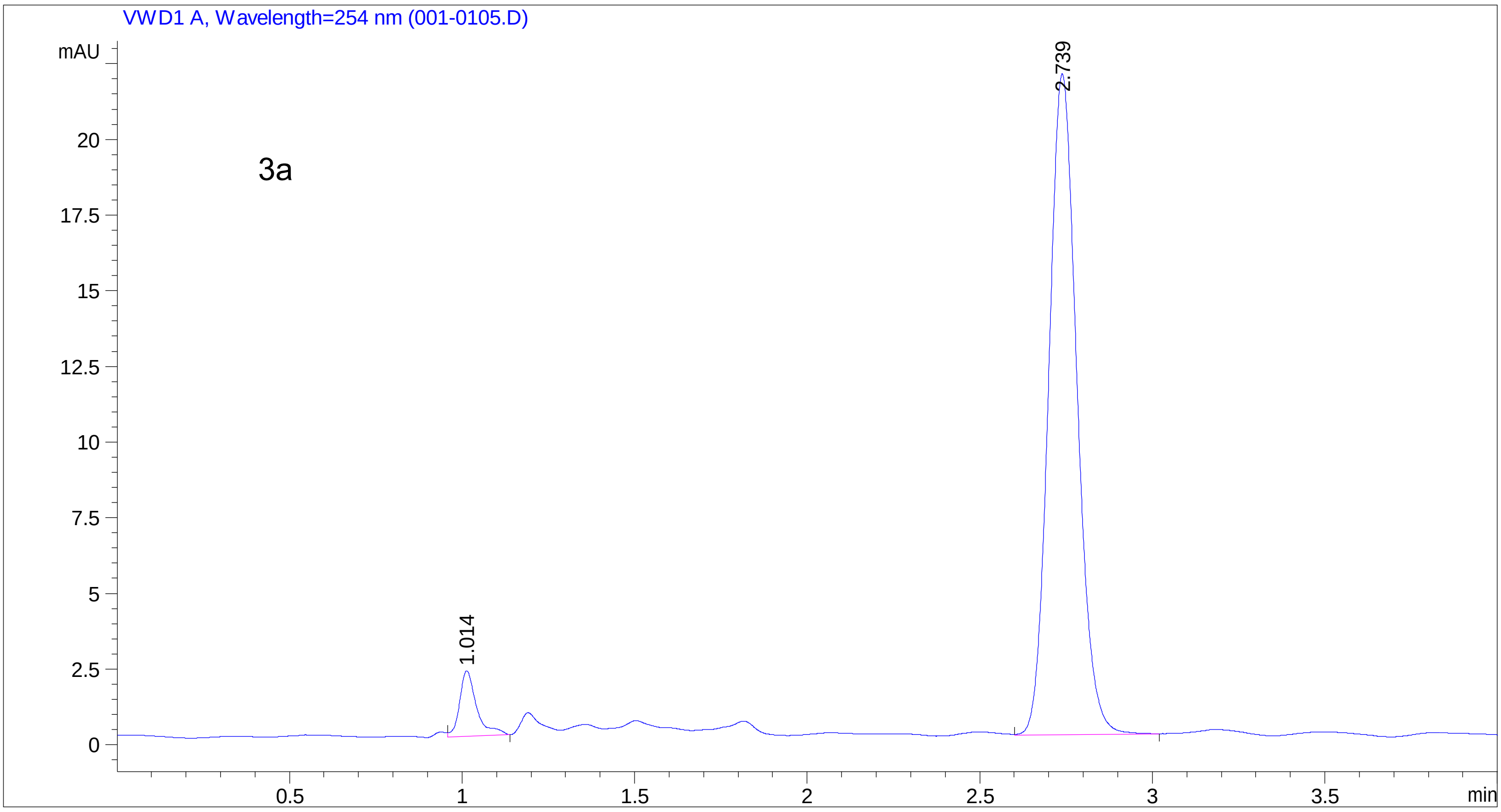

===

Area Percent Report

===

Sorted By : Signal
Multiplier : 1.0000
Dilution : 1.0000
Use Multiplier & Dilution Factor with ISTDs

Signal 1: VWD1 A, Wavelength=254 nm

Peak #	RetTime [min]	Type	Width [min]	Area [mAU*s]	Height [mAU]	Area %
1	1.014	VB	0.0512	7.45310	2.16099	5.6793
2	2.739	VB	0.0876	123.78002	21.83794	94.3207
Totals :				131.23313	23.99892	

===

*** End of Report ***

Data File C:\CHEM32\1\DATA\001-0110.D
Sample Name:

===

Acq. Operator : SYSTEM Seq. Line : 1
Acq. Instrument : LC Location : Vial 1
Injection Date : 7/22/2026 1:14:44 PM Inj : 10
Inj Volume : Manually
Sequence File : C:\CHEM32\1\SEQUENCE\KEM.S
Method : C:\CHEM32\1\METHODS\KAM.M
Last changed : 7/22/2026 1:00:56 PM by SYSTEM
(modified after loading)

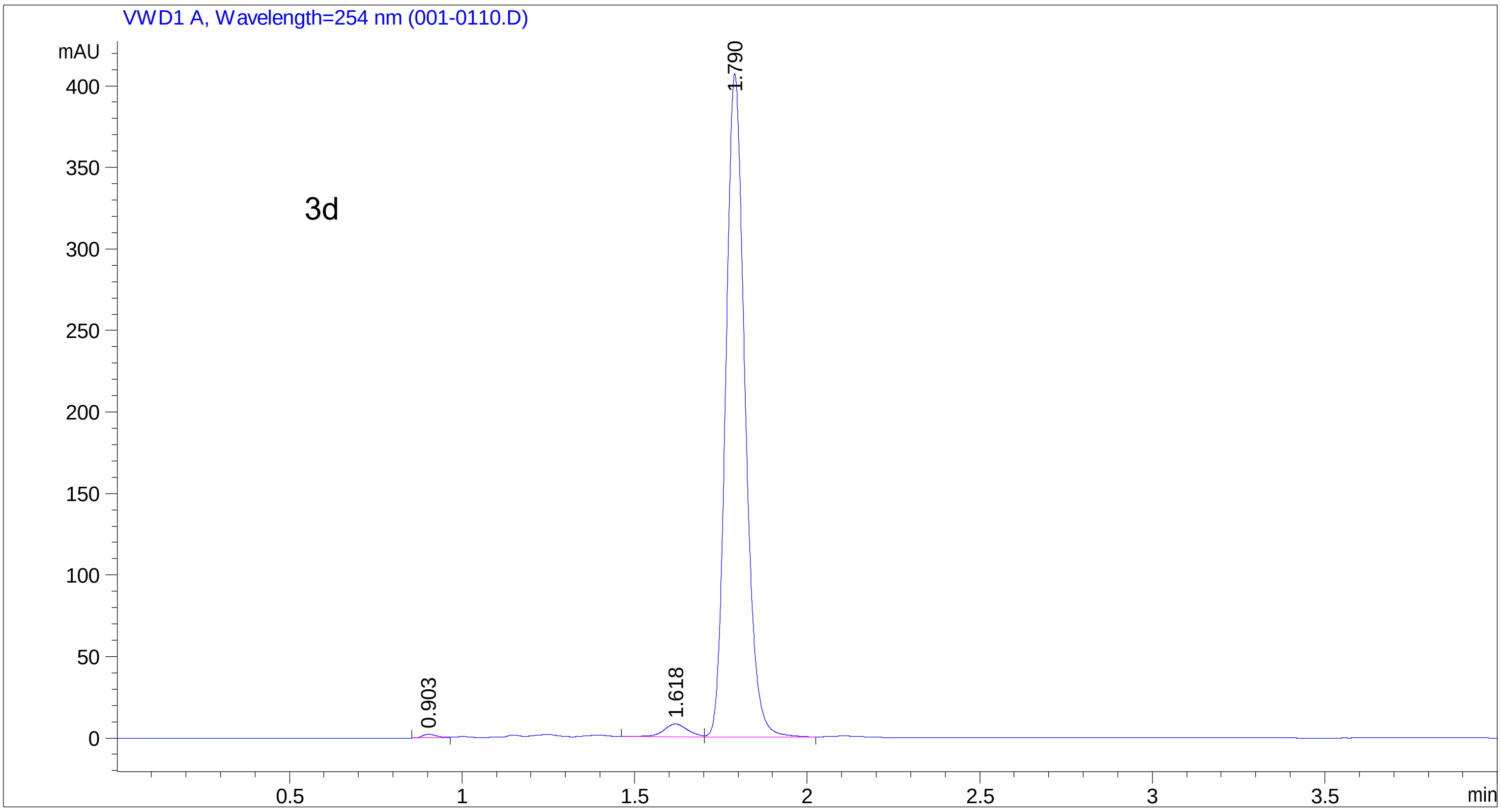

===

Area Percent Report

===

Sorted By : Signal
Multiplier : 1.0000
Dilution : 1.0000
Use Multiplier & Dilution Factor with ISTDs

Signal 1: VWD1 A, Wavelength=254 nm

Peak #	RetTime [min]	Type	Width [min]	Area [mAU*s]	Height [mAU]	Area %
1	0.903	BV	0.0442	6.68037	2.27947	0.4122
2	1.618	BV	0.0732	38.35580	7.89205	2.3666
3	1.790	VV	0.0602	1575.68213	406.72134	97.2212

Totals : 1620.71829 416.89286

===

*** End of Report ***

Data File C:\CHEM32\1\DATA\001-0108.D
Sample Name:

===

Acq. Operator : SYSTEM Seq. Line : 1
Acq. Instrument : LC Location : Vial 1
Injection Date : 6/16/2026 2:16:36 PM Inj : 8
Inj Volume : Manually
Sequence File : C:\CHEM32\1\SEQUENCE\KEM.S
Method : C:\CHEM32\1\METHODS\KAM.M
Last changed : 6/16/2026 2:17:56 PM by SYSTEM
(modified after loading)

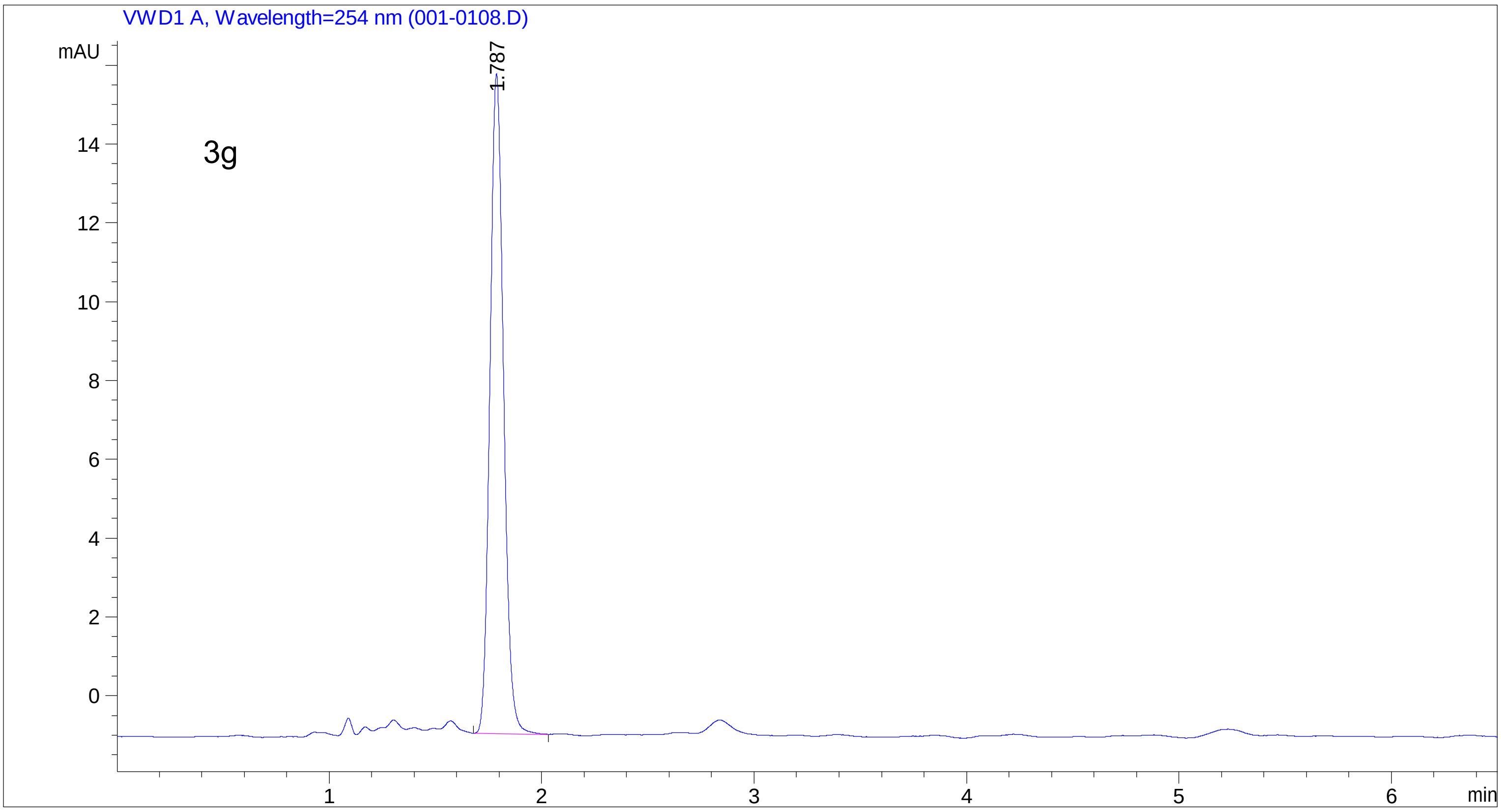

===

Area Percent Report

===

Sorted By : Signal
Multiplier : 1.0000
Dilution : 1.0000
Use Multiplier & Dilution Factor with ISTDs

Signal 1: VWD1 A, Wavelength=254 nm

Peak #	RetTime [min]	Type	Width [min]	Area [mAU*s]	Height [mAU]	Area %
1	1.787	BB	0.0688	74.36150	16.74156	100.0000

Totals : 74.36150 16.74156

===

*** End of Report ***
